# Coupling Exponential to Linear Amplification for Endpoint Quantitative Analysis

**DOI:** 10.1002/advs.202309386

**Published:** 2024-04-09

**Authors:** Coline Kieffer, Yannick Rondelez, Guillaume Gines

**Affiliations:** ^1^ Laboratoire Gulliver UMR7083 CNRS/ESPCI Paris‐PSL Research University 10 rue Vauquelin Paris 75005 France

**Keywords:** CELIA, DNA circuits, DNA nanotechnology, endpoint readout, isothermal amplification, microRNAs, nucleic acid amplification

## Abstract

Exponential DNA amplification techniques are fundamental in ultrasensitive molecular diagnostics. These systems offer a wide dynamic range, but the quantification requires real‐time monitoring of the amplification reaction. Linear amplification schemes, despite their limited sensitivity, can achieve quantitative measurement from a single end‐point readout, suitable for low‐cost, point‐of‐care, or massive testing. Reconciling the sensitivity of exponential amplification with the simplicity of end‐point readout would thus break through a major design dilemma and open a route to a new generation of massively scalable quantitative bioassays. Here a hybrid nucleic acid‐based circuit design is introduced to compute a logarithmic function, therefore providing a wide dynamic range based on a single end‐point measurement. CELIA (Coupling Exponential amplification reaction to LInear Amplification) exploits a versatile biochemical circuit architecture to couple a tunable linear amplification stage – optionally embedding an inverter function – downstream of an exponential module in a one‐pot format. Applied to the detection of microRNAs, CELIA provides a limit of detection in the femtomolar range and a dynamic range of six decades. This isothermal approach bypasses thermocyclers without compromising sensitivity, thereby opening the way to applications in various diagnostic assays, and providing a simplified, cost‐efficient, and high throughput solution for quantitative nucleic acid analysis.

## Graphical abstract

CELIA, a hybrid DNA/enzyme circuit combining the sensitivity and broad dynamic range of exponential nucleic acid amplification with simple and cost‐efficient end‐point readout is presented. Applied to microRNA quantification, CELIA achieves subpicomolar limit of detection over six decades. Its isothermal nature obviates the need for thermocyclers while maintaining sensitivity, enabling its use in diverse diagnostic assays.

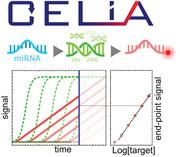

## 1 Introduction

**Figure 1:**
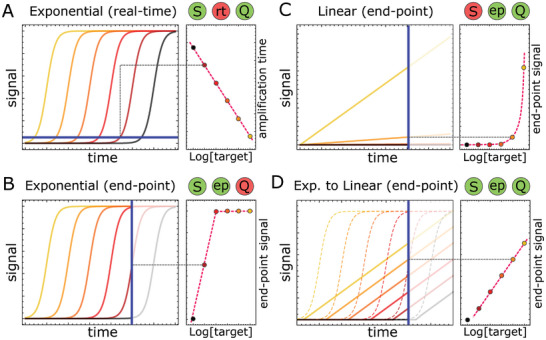
Comparison of the sensitivity (S), quantitativity (Q) and readout (real‐time, rt or endpoint, ep) for various amplification schemes, applied to a serial dilution of a target. A) Exponential amplification enables highly sensitive target detection, but it requires the real‐time monitoring of the amplification reaction to provide quantitative information. B) Exponential amplification reactions with endpoint analysis provide low‐cost and fast readout, but the saturation of the signal occurs too rapidly to retrieve quantitative information, rendering it suitable only for qualitative analysis C) Linear amplification can be used for endpoint target quantification, however with a limited sensitivity due to the low amplification fold. D) The proposed CELIA approach combines the sensitivity of exponential amplification with the convenience and lower cost of endpoint readout afforded by linear amplification. This is achieved by conditionally implementing a linear amplification stage after the exponential amplifier stage. Horizontal and vertical blue lines correspond to the selected amplification signal threshold and endpoint time, respectively.

Exponential DNA amplification methods are a cornerstone of ultrasensitive molecular diagnostics. Although polymerase chain reaction (PCR) is the most emblematic illustration^[^ ^1^, ^2^ ^]^, isothermal amplification schemes are rapidly expanding, offering promising alternatives. For example, loop‐mediated isothermal amplification (LAMP, used notably for rapid SARS CoV‐2 testing^[^ ^3^ ^]^), exponential rolling circle amplification (eRCA, that leverages processive and strand‐displacing enzymes like Phi29 DNA polymerase),^[^ ^4^ ^]^ or Exponential Amplification reaction (EXPAR, which can be easily connected to diverse input targets like DNA, RNA, protein or even small metabolites)^[^ ^5^ ^]^ allow exponential process going from a few copies of a nucleic acid target, up to easily detectable concentrations – typically via an optical readout. Quantification measurements, however, rely on the real‐time kinetic monitoring of the reaction to extract an amplification time (e.g. cycle threshold, Ct for qPCR), which is logarithmically related to the initial target concentration (**Figure** **1**A). As such, these exponential assays offer an exceptional dynamic range (up to 8 decades).^[^ ^6^ ^]^ Although a simpler, endpoint measurement is possible, it drastically reduces the dynamic range^[^ ^7^ ^]^ and is generally only used to retrieve qualitative (yes/no) information,^[^ ^8^ ^]^ essentially because the exponentially amplified signal quickly reaches saturation (Figure 1B).

By contrast, linear amplification schemes such as RCA^[^ ^9^, ^10^ ^]^ or strand displacement amplification (SDA)^[^ ^11^ ^]^ authorize quantitative endpoint readout. However, owing to the slow, linear accumulation of signal, these methods are much less sensitive than their exponential counterparts and provide a linear dynamic range (Figure 1C).

In this work, we design a modular DNA/enzyme‐based reaction network that allows for quantitative endpoint measurement of nucleic acid targets while maintaining the sensitivity and wide dynamic range of an exponential strategy. The concept relies on Coupling an Exponential amplification reaction to LInear Amplification stage with tunable gain (CELIA, Figure 1D).

## 2 Results and Discussion

We built on a previously reported EXPAR‐like molecular switch based on the polymerase‐exonuclease‐nickase (PEN) toolbox,^[^ ^12^, ^13^ ^]^ which allows exponential signal strand amplification while mitigating nonspecific reactions^[^ ^14^ ^]^. The original circuit is assembled from three DNA templates: the amplification template (aTα) is a dual‐repeat sequence embedding a nicking recognition site, which encodes the exponential replication of a signal strand (α) upon polymerization/nicking cycle; the pseudo‐template (pTα) is made of the complementary sequence of α to which is appended a short 5’ polynucleotide sequence (e.g. pentathymidylate) that allows the deactivation of α by polymerase extension;^[^ ^14^ ^]^ the reporter template (rTα) is a hairpin‐shape structure labeled with a fluorophore and a quencher, that is used to probe the presence of complementary α strands. To implement a CELIA circuit, we connected this exponential amplification module to the linear production of a secondary signal strand (ω) by introducing two additional templates. The first one is a linear template (αtoω) that takes α as input and creates ω via polymerization/nicking cycles. The second one is a ω‐specific reporter template (rTω) which reveals the accumulation of ω in a second fluorescent channel.

**Figure 2:**
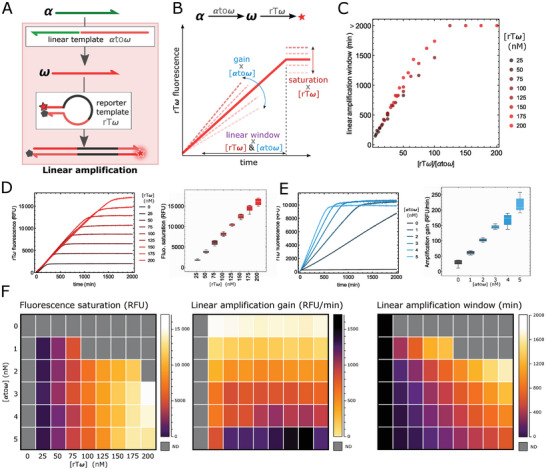
A linear amplification module with tunable response. A) The module is composed of the template αtoω, which linearly produces ω once loaded with the α input, and rTω, which generates a fluorescence signal once bound to ω. B) The linear amplification is monitored in real‐time for different concentrations of the two templates, allowing us to extract the amplification gain (related to the slope), the saturation level, and the linear response time window (i.e. the time it takes for the signal to reach saturation). C) Linear amplification window as a function of the ratio [rTω] /[αtoω]. D) Linear amplification curves for [αtoω] = 5 nm and various [rTω]. The right plot corresponds to box and whisker chart of the rTω saturation level as a function of [rTω] for various [αtoω]. E) Linear amplification curves for [rTω] = 125 nm and various [αtoω]. The right plot corresponds to box and whisker chart of the amplification gain as a function of [αtoω] for various [rTω]. F) Color‐coded array plots of the fluorescence saturation (left), amplification gain (middle), and linear amplification window (right).

We first tested the linear amplification module in isolation (see Figures S1–S5 (Supporting Information) for detailed reaction networks). We preloaded αtoω with an excess of its input and monitored the signal generated by the red‐fluorescent reporter rTω (**Figure** **2A**). We indeed observed a linear response in time followed by a saturation phase when all rTω has reacted. Additionally, the response of this amplifier can be finely tuned in terms of i) the amplification gain, by varying the concentration of αtoω (Figure 2E,F); ii) the signal saturation, which is directly proportional to the concentration of rTω (Figure 2D,F); iii) the linear amplification time window (before reaching saturation), which depends on the ratio between αtoω and rTω (Figure 2C,F).

**Figure 3:**
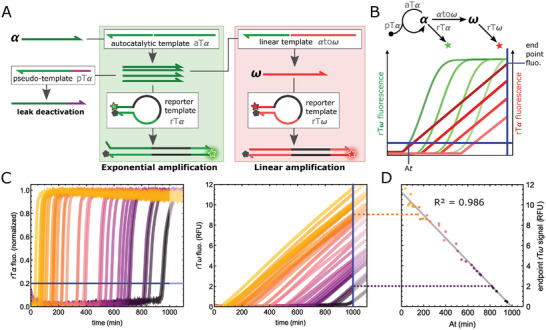
Negative linear correlation between the amplification time of the exponential module and the endpoint signal of the linear module. A) The linear module is connected downstream of an exponential amplification reaction, whose start time is controlled by the initial concentration of α and pTα. B) Both amplifications are monitored in parallel through the corresponding rT fluorescence, allowing the extraction of amplification times (rTα) and endpoint signals (rTω). C) Time traces for the exponential (left) and linear amplification (right). Matching colors on the two plots correspond to the same sample (see also Figure S6, Supporting Information). The horizontal blue line materializes the signal threshold used to extract the amplification time (At) in the exponential mode. The vertical blue line corresponds to the endpoint time in the linear amplification mode. D) Endpoint rTω (t = 1000 min) as a function of the amplification time At.

We next connected the linear amplification module downstream of an exponential amplification switch (aTα/pTα/rTα) in such a way that the endpoint signal from the linear amplifier (rTω fluorescence) should be inversely correlated to the amplification time of the exponential switch (At, i.e. the time it takes for the rTα fluorescence to rise above a given detectable value, **Figure** **3**A and B). To check this correlation, we continuously modulated the At values between 10 and 1000 min by varying the initial concentration of α initiator (the higher, the sooner the At) and pTα inhibitor (the higher, the later the At,^[^ ^14^ ^]^ Figure 3C left). The red (rTω) fluorescence signal (Figure 3C right) reveals that the linear stage quasi‐instantaneously kicks in following the α amplification module, and rapidly reaches a linear regime (see also Figure S6B, Supporting Information). In this experiment, the αtoω and rTω concentrations (2 and 200 nm respectively) were set so that the linear amplification window is larger than 1000 min, corresponding to the range of tested amplification times. We recorded the endpoint red (rTω) fluorescence, which, as expected, is negatively correlated to the At, with a coefficient of determination R^2^ = 0.986 (Figure 3D). The correlation can be improved by normalizing the rTω with the fluorescence at saturation (Figure S7, Supporting Information). This suggests that the slight deviation from the linear regression curve can be attributed in part to inter‐well variations of the optical signal recorded by the real‐time fluorescence scanner and would be mitigated with an appropriate fluorescent setup. Importantly, we verified that the linear amplification gain is consistent over time (1.15 10^−2^ ± 5.9 10^−4^ RFU min^−1^, CV ≈ 5%) and independent from the exponential amplification time (Figure S6E, Supporting Information). This is a crucial requirement for being able to use the endpoint rTω signal in quantitative biosensing applications.

**Figure 4:**
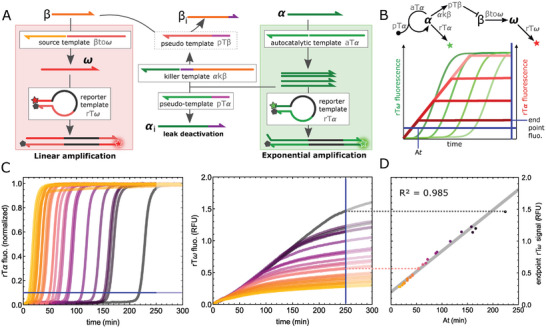
Inverter function. A) The circuit is composed of an exponential amplification module (aTα, pTα and rTα) and a constitutive linear amplification module (βtoω, β and rTω) interconnected by the killer template αkβ. Upon amplification of the α signal, αkβ produces pTβ, which in turn depletes the pool of β strand. This makes the linear amplification stop. As a result, the sooner the exponential amplification, the lower the endpoint linear amplification signal. B) Both amplifications are monitored in parallel through the corresponding rT fluorescence, allowing the extraction of amplification times (rTα) and endpoint signals (rTω). C) Time traces for the exponential (left) and linear amplification (right). Matching colors on both graphs correspond to the same samples (see also Figure S11, Supporting Information). The horizontal blue line depicts the signal threshold for extracting the amplification time (At) in the exponential mode and the vertical blue line corresponds to the endpoint chosen in the linear amplification mode. D) Endpoint rTω as a function of the amplification time. The data points are fitted with linear regression.

Reversing the signal to obtain a positive correlation between the amplification time and the end‐point rTω signal would enhance the modularity of the molecular circuit, enabling the implementation of logical operations (e.g., NOT gate), which are fundamental in building complex molecular programming systems.^[^ ^15^, ^16^, ^17^ ^]^ The circuit architecture has been adjusted to encode such inverter function (**Figure** **4**A): a converter template βtoω constitutively produces ω at a rate controlled by the concentration of βtoω and its input β (Figure S8, Supporting Information). This linear amplification module hence generates a linear increase in rTω fluorescence from the beginning of the incubation. Once the exponential amplification of α kicks in, it triggers the production of a β inhibitor under the form of a pseudotemplate (pTβ) via the killer template αkβ^[^ ^18^ ^]^ (which uses α as input and produces pTβ as output). Since β reversibly binds to βtoω (Experimental Section, see the section on template design), it can hybridize to the newly produced pTβ, and be extended by the DNA polymerase which releases an inactive form (β_i_). This stops the production of ω and eventually makes the rTω signal plateau. Figure 4C shows the real‐time amplification curves of α (exponential) and ω (linear). Like in the experiment shown in Figure 3, the α At is modulated by the pTα inhibitor and the α initiator concentration. As anticipated, the sooner the α amplification, the lower the ω plateau. The endpoint ω signal is now positively correlated to the α amplification time (R^2^ = 0.985, Figure 4D). We noticed that the inhibition of the linear amplification occurs after a certain delay from the exponential amplification time, which is dependent on the linear amplification gain (i.e., the concentration of β and βtoω, Figure S9, Supporting Information) and can be reduced by increasing the concentration of αkβ. However, increasing αkβ also aggravates the nonspecific production of pTβ stemming from the leak of the killer template (Figure S10, Supporting Information). Such a leak is responsible for the premature decrease of the ω production rate and results in an underestimation of the endpoint ω signal at high amplification time (> 250 min for αkβ = 1 nm).

**Figure 5:**
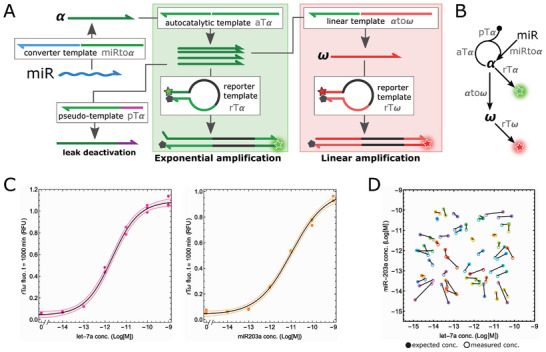
Endpoint readout applied to miRNA detection. A) Schematic of the miRNA endpoint sensing circuit. A miRNA converter template (miRtoα) is connected upstream to the exponential bistable module. B) Simplified scheme of the circuit architecture. C) Calibration curve for triplicated samples containing various concentration of let‐7a (left) and miR‐203a (right). The data points are fitted with a simple sigmoidal curve (black curve). The shaded area corresponds to the 90% confidence interval over the fitted parameters. D) Analysis of synthetic samples spiked with random concentrations of let‐7a and miR‐203a (from 10^−14^ M to 10^−10^ M).

The CELIA system was applied to the end‐point quantification of microRNA (miRNA) biomarkers.^[^ ^19^ ^]^ We connected the exponential‐to‐linear amplification circuit (with no inverter function) circuit downstream of a converter template (miRtoα) that transduces the presence of an input miRNA into the generation of α strands (**Figure** **5**A,B).^[^ ^20^ ^]^ In this configuration, the higher the target miRNA concentration, the sooner the amplification, hence the higher the endpoint rTω fluorescence signal. The assay was calibrated for two miRNAs, let‐7a and miR‐203a, both of which have been demonstrated to be associated with cancer diseases.^[^ ^21^, ^22^ ^]^ The amplification mixture was spiked with a varying concentration of either of these miRNAs and the endpoint fluorescence of rTω (t = 1000 min) is reported in Figure 5C. As expected, the endpoint signal is positively correlated to the miRNA concentration over 6 orders of magnitude, with a limit of detection (LoD, determined from the mean signal of the negative control plus 3 times the standard deviation) of 17 and 33 fm for let‐7a and miR‐203a, respectively. These values are in agreement with the LoD computed from the exponential amplification (28 and 16 fm, respectively, Figure S12, Supporting Information), demonstrating that the digital‐to‐analog signal conversion does not negatively impact the sensitivity. In addition, a simple linear amplification scheme (i.e. in the absence of any nonlinear signal amplification) displays a LoD of 8.5 pm (Figure S13, Supporting Information), supporting the gain of sensitivity brought by our exponential‐to‐linear amplification strategy, although optimized RT‐qPCR assays displayed lower LoD (0.70 and 0.12 fm for let‐7a and miR‐203a, respectively (Figure S14, Supporting Information).

Leveraging miRNAs as biomarkers generally requires ultrasensitive detection, and thus real‐time techniques.^[^ ^23^ ^]^ The sample throughput is therefore limited by the immobilization of costly optical apparatus (e.g. 96‐well qPCR machines) over the full duration of the assay. In the case of endpoint readout, on the contrary, many reactions can be incubated together offline, and then quickly analyzed in a sequential manner by a simple optical reader. To demonstrate that endpoint CELIA can indeed help mitigate the sample bottleneck in ultrasensitive diagnosis without compromising the quality of the measurement, we prepared 54 synthetic samples containing two clinically‐relevant miRNAs (let‐7a and miR‐203a) at two different concentrations randomly taken from a uniform distribution on the interval 5 fm–100 pm in logarithmic scale. Each miRNA of each sample was then individually assayed by using the cognate converter template in a CELIA reaction, and the endpoint rTω fluorescence was recorded. The experimental concentrations were then computed with reference to a calibration curve (Figure 5C). The two measured concentrations in each sample were very close to the expected pattern of spiked concentrations, with an average fold difference of 2.3 ± 1.4 and 2.1 ± 1.9 for let‐7a and miR‐203a, respectively (Figure 5D). Importantly, the real‐time readout, performed in the same experiment, displays a similar, although slightly higher accuracy (1.8 ± 1.3 and 1.5 ± 0.7 fold difference for let‐7a and miR‐203a, respectively(Figure S15, Supporting Information). However, we observed that when both miRNAs were present, the qPCR assay for miR‐203a lacked specificity, resulting in a strong quantification bias (Figure S16, Supporting Information).

## 3 CELIA Circuit Design

In previously described isothermal exponential amplification schemes, real‐time monitoring of the reaction is required to extract the amplification time, which linearly correlates with the log‐concentration of the target (the higher the concentration, the sooner the amplification). Linear amplification schemes offer the advantage of simpler, endpoint readout, at the cost of a lower sensitivity. For instance, the linear signal amplification provided by the collateral activity of CRISPR/Cas systems (e.g. Cas12a or Cas13a) has recently attracted considerable attention in diagnostics.^[^ ^24^, ^25^, ^26^ ^]^ However, CRISPR detection schemes are often combined with a target pre‐amplification step to increase their sensitivity to clinically‐relevant levels.^[^ ^27^, ^28^, ^29^ ^]^ In this work, we described a 1‐step DNA/enzyme‐based amplification circuit that converts the digital mode of a saturable exponential amplification to an analog regime through an additional linear amplification layer. The linear module gradually releases a fluorescent signal, thereby allowing ultrasensitive and wide dynamic range measurement via a single endpoint record. We demonstrated that the gain, tunable by simply varying the concentration of one template, is constant over time and does not depend on the amplification time, which indicates the robustness of the circuit and enzymes to experimental conditions. This constant gain is essential to preserve the quantitative nature of the real‐time assay and to translate it to a simpler endpoint readout.

In addition, we provided the circuit with an inverter function that allows to reverse the correlation between amplification time and end‐point signal. Such a feature would be of interest to transpose the assay to a simple and cost‐efficient visual readout (e.g. lateral flow assay or other colorimetric formats) for which a positive correlation facilitates the interpretability and intuitive understanding of the results. In the context of DNA computing, and more widely synthetic biology, such inverter (or NOT gate), where the input molecule induces a repression of the output molecule, is an essential component of – synthetic – gene regulatory networks, which can implement a wide range of dynamics.^[^ ^30^, ^31^ ^]^ We envision in the future the implementation of the presented circuit for neuromorphic molecular computing, which requires analog input integration and both positive and negative correlations.^[^ ^17^, ^32^, ^33^, ^34^, ^35^ ^]^

**Figure 6:**
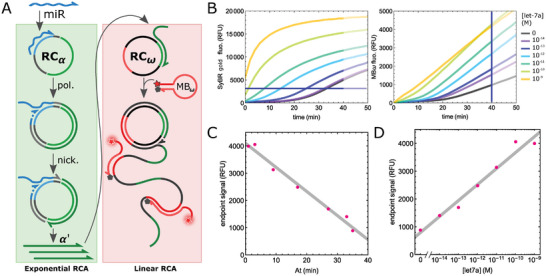
RCelia for sensitive and quantitative endpoint RCA. A) An exponential RCA is triggered by the miRNA target (let‐7a). The nicking of the RCA product generates short DNA fragments that are used to load other circular templates. The output α’ of the exponential RCA initiates a secondary linear RCA on the circular template RCω. This RCA product hybridizes to a pro‐fluorescent molecular beacon MBω that reports the progress of the reaction. B) Time traces for various let‐7a concentrations of the exponential amplification (left, monitored with a double strand specific dye SYBR green) and linear amplification (monitored via MBω fluorescence). It can be noted that the RCelia exponential amplification efficacy as well as the linear amplification gain decrease as the amplification increases. However, the linear stage reaches constant velocity following a short acceleration phase. These observations suggest a decrease in the enzymatic activity of the nicking enzyme essential to the exponential replication of α’, but not required in the linear phase. C) Endpoint MBω fluorescence as a function of At. D) Endpoint signal as a function of the let‐7a target concentration.

Our strategy of combining exponential and linear amplification modules to produce logarithmic responses in the concentration – not time – domain is in fact a general strategy that has already been proposed in other contexts.^[^ ^36^ ^]^ Sanchez *et al.* reported an asymmetric implementation of PCR where the exponential replication of the double‐stranded target sequence is followed by a linear production of single‐stranded DNA from the primer in excess (LATE‐PCR).^[^ ^37^ ^]^ However, these efforts were developed in an attempt to improve real‐time fluorescence reporting and were not leveraged for quantitative end‐point protocols. To further support its usefulness and relevance in the field of ultrasensitive diagnostics, we tested the possibility of applying the exponential‐to‐linear strategy to other isothermal amplification primitives, such as the rolling circle amplification (RCA) reaction. We thus assembled the RCelia scheme, in which a nickase‐assisted exponential RCA reaction^[^ ^38^ ^]^ is combined with a second linear‐only RCA (**Figure** **6**A). The exponential RCA is initialized by the target miRNA (let‐7a) and produces a short linear output (α’) by DNA polymerization and nicking along the circular probe RCα. α’ initiates the linear RCA along the second circular probe (RCω). Similar to the presented DNA circuit, while α’ is exponentially amplified up to a saturating level (monitored with the double‐strand specific dye SYBR Gold), the downstream reaction restores a signal linearly correlated to the logarithm of the miRNA concentration, enabling endpoint, quantitative measurement, with a limit of detection in the sub‐picomolar range, constrained by the nonspecific initiation of the RCA reaction (Figure 6B–D).^[^ ^39^ ^]^

Such quantitative endpoint readout mode would significantly reduce the burden of optical instrument run time and improve sample throughput. For instance, a typical RT‐qPCR assay for miRNA quantification, performed in a 96‐well plate format, requires 2 h of real‐time thermocycling machine, leading to a maximum of 1152 samples in 24 h without delay between assays. By switching to an endpoint readout, the incubation step can be performed at a large scale (e.g. in a low‐cost incubator) while the optical instruments are only used for the post‐incubation measurement. With a 2 min turnover between measurements, the throughput can be increased by a factor of 60, allowing the testing of close to 70 000 samples per day.

## 4 Experimental Section

### Materials

High‐performance liquid chromatography‐purified oligonucleotides were purchased from Biomers or Eurofins and resuspended at 100 µm in 1X Tris−EDTA at pH 7.5 for long‐term storage. The enzymes Nb.BsmI, Nb.BbvCI, Nb.BssSI, Nt.BstNBI, BsmI, DNA polymerase Vent (exo‐), BSA (200 µg.mL^−1^) and dNTPs were obtained from New England Biolabs (NEB). A tenfold dilution of Nt.BstNBI was prepared in Diluent A supplemented 0.1% (v/v) Triton X100, and stored as the other enzymes at −20 °C. The CircLigase ssDNA was received from Biosearch Technologies. Phi29 DNA polymerase, Phi29 DNA polymerase reaction buffer 10X, DTT and SYBR Gold Nucleic Acid Gel Stain 10 000X were purchased from ThermoFisher. Thermus thermophilus RecJ exonuclease was produced in‐house by following a previously published protocol^[^ ^40^ ^]^ and was diluted by 140 in Diluent A (NEB) supplemented 0.1% (v/v) Triton X100 (Merk). Sodium chloride, potassium chloride, magnesium sulfate, ammonium sulfate, Trizma hydrochloride, netropsin, and synperonic F104 were purchased from Merck (Sigma–Aldrich). All solutions were prepared using ultrapure water from a Millipore Milli‐Q water purification system. Ligation and digestion reactions for RCA experiments were performed in an Eppendorf ThermoMixer C. All reactions presented in this paper were monitored with a CFX96 Touch Real‐Time PCR machine (Bio‐Rad) and the results were analyzed with Mathematica software (Wolfram).

### PEN‐DNA Template Design

Templates were designed according to the rules described elsewhere.^[^ ^13^, ^18^, ^41^ ^]^ Template sequences α, pTα, rTα, αtoω, βtoω, αkβ, and rTω were protected against the 5′ → 3′ exonuclease activity of ttRecJ by the addition of three or four 5′ phosphorothioate backbone modifications. Templates cTα, aTα, pTα αtoω, βtoω, and αkβ were blocked to prevent unwanted polymerization by the addition of a 3′ phosphate moiety. The α input binding site of aTα was shortened by 2 bases to hybridize only to the last 10 bases of the α strand. This was to favor the deactivation by pTα of α strands stemming from leaky reactions on aTα (resulting for instance from ab initio synthesis by the DNA polymerase). αtoω ssDNA was designed to hybridize only the 9 3’ bases of α to minimize its competition with the autocatalytic template. The activation of αtoω was made irreversible using a type IIS nicking site (Nt.BstNBI). In the case of the inverter function, the activation of the βtoω template for ω production needs to be reversible to allow its inhibition by the killer template αkβ. To do so, the template contains a type IIP nicking site (Nb.BssSI) that is cleaved one base away from the 5’ recognition sequence of the nickase. Cleavage of βtoω by Nb.BsssI releases an ω with a 5’ overhang corresponding to the recognition sequence, which does not affect the binding to and extension along rTω. More importantly, the cleavage leaves a single additional 3’ C on β, so that this modified input was still able to unbind from βtoω and be deactivated by the output of αkβ, hence permitting the blockage of ω production. Note that the output of αkβ produces pTβ, a degradable pseudotemplate that can deactivate both β and its 3’ C‐extended version. See Table S1 (Supporting information) for a detailed list of the sequences used in this study and Figure S17 (Supporting information) for a schematic representation.

### PEN‐DNA Reaction Assembly

All reactions were assembled on ice. Ultrapure milliQ water and 4X reaction buffer (80 mm tris‐HCl (pH 8.9), 40 mm (NH4)_2_SO4, 160 mm KCl, 40 mm MgSO4, 100 µm each dNTP, 0.4% (w/v) Synperonic F108, 8 µm netropsin) were mixed, followed by the addition of the templates, BSA (200 µg.mL^−1^) and the enzymes (cf. Figure S18 (Supporting information) for exact sample composition). For miRNA detection, serial dilutions of a synthetic version of let‐7a were performed on parafilm in Tris‐EDTA 1X supplemented with 1 nm of pTα to minimize target carry‐over. The dilution was executed using the pipet and mix mode of the electronic pipette Xplorer (Eppendorf), using a low binding DNA epDualfilter T.I.P.S. 1 – 20 µL (Eppendorf, cat# 0030078500).

### Synthetic miRNA Samples

Pairs of concentrations of let‐7a and miR‐203a were randomly selected in the logarithmic domain from the interval 10^−14^–10^−12^ m. Sample assembly was performed on an automated liquid handling robot (I‐DOT Deepwell, Dispendix) in 384‐well plate (Bio‐Rad), assisted by a custom‐made script. Each sample was duplicated and spiked with either of the converter templates (let7atoα or 203atoα). In addition, a calibration curve for each individual miRNA was realized with triplicate samples of a tenfold dilution of miRNA (from 10^−9^ to 10^−15^ m, including a no miRNA control). The standard data points were fitted with a sigmoid function: $f\;\left(x\right)=\;L+H/\left(1+b.e^{-k(x-x_{0})}\right)$, where f(x) is the output (y‐value) of the sigmoid function at input *x*, *L* and *H* are respectively the lower and higher bound (*L* = lower and *H*  +  *L* = upper asymptote), *k* is the steepness of the curve and *x*_0_ the x‐value at mid‐point of the sigmoid.

### RCA Template Design

RCα ssDNA template was designed to bind let‐7a as an input. The RCα sequence contains two recognition sites for Nb.BbvCI such that each polymerization/nicking cycle releases one α’ strand and one secondary strand that were both released by polymerase‐mediated strand displacement and bind free RCα. As a result, the target miRNA could trigger an initial exponential amplification of α’.  Subsequently, α’ partially binds RCω and initiates the linear amplification stage. Note that RCω templates did not contain any nicking site. The resulting polymerization upon RCω generated a long ssDNA concatemer that bound and opened the stem of the molecular beacon (MBω). The loop of this hairpin‐shaped molecular beacon has been designed to bind the output of the linear RCA template over 28 bases and to release fluorescence upon its hybridization at 37 °C. MBω was conjugated with a Cyanine 5 fluorophore at the 5’ extremity and with a BHQ‐2 quencher at the 3’ extremity. The stem composed of 8 bp and three 2’O‐methyl uracil was added to the 3’ end to reinforce the stem structure at 37 °C.

### Circular Template Synthesis and Purification

The ssDNA oligonucleotides were circularized with the CircLigase ssDNA (Biosearch Technologies). This enzyme is a thermostable ATP‐dependent ligase that catalyzes the intramolecular ligation of ssDNA templates having 5´‐phosphate and 3´‐hydroxyl group at their extremities. The ligation reaction was performed in 1X Ligase reaction buffer from the provider, 50 µm ATP, 2.5 mm MnCl_2_, 100 units CircLigase ssDNA, 1 µm ssDNA template and supplemented with nuclease‐free water to adjust the volume to 10 µL. The ligation reaction mixture was finally heated at 60&nbsp;°C for 2 h. The ligated products were subsequently treated with exonuclease I to remove unligated linear templates in 1X rCutSmart Buffer (NEB), 2 U.µL^−1^ of Thermolabile exonuclease I and 500 nm of ligated products. The digestion was pursued for 2 h at 37°C and the Exo I was finally deactivated at 80°C for 10 min. Samples were finally heated at 95°C for 10 min to unfold possible secondary structures of circular ssDNA templates, which were quantified by fluorometry using the Qubit 4 nucleic acid quantification (Invitrogen).

### RCA Reaction

All reactions were assembled on ice. Ultrapure milliQ water and 10X Phi29 DNA polymerase reaction buffer (330 mm Tris‐acetate (pH 7.9 at 37°C), 100 mm Mg‐acetate, 660 mm K‐acetate, 1% (v/v) Tween 20, 10 mm DTT) were mixed, followed by the addition of the two circular templates, 1 mm dNTPs, BSA (600 µg.mL^−1^), 1.25X SYBR Gold and the necessary enzymes (cf. Figure S18 (Supporting Information) for exact sample composition). Samples were finally heated at 37°C to perform the exponential‐to‐linear reaction. The miRNA dilution protocol was identical to the PEN‐DNA reaction described above.

### RT‐qPCR Two‐Step Procedure

All reactions were assembled on ice. Target dilutions were performed on parafilm for the PEN‐DNA reaction assembly section. The RT step was prepared by using the TaqMan MicroRNA Reverse Transcription Kit (cat #4366596, lot n°2888473) supplemented with the TaqMan microRNA assay for let‐7a and miR‐203a (cat #4427975). The qPCR step was performed with the TaqMan Universal Master Mix II, no UNG (cat #4440043, lot n°2768394) from ThermoFisher and with the PCR primers provided in the TaqMan microRNA assays. The entire RT‐qPCR procedure was executed by following the protocol recommended by the provider. PCR thermocycling was performed in a CFX96 Touch Real‐Time PCR machine (Bio‐Rad) and the results were analyzed with Mathematica software (Wolfram).

## Conflict of Interest

The authors declare no conflict of interest.

## Supporting information

Supporting Information

## Data Availability

The data that support the findings of this study are available from the corresponding author upon reasonable request.

## References

[1] H. Zhu , H. Zhang , Y. Xu , S. Laššáková , M. Korabečná , P. Neužil , BioTechniques. 2020, 69, 317. 32815744 10.2144/btn-2020-0057 PMC7439763

[2] M. Li , F. Yin , L. Song , X. Mao , F. Li , C. Fan , X. Zuo , Q. Xia , Chem. Rev. 2021, 121, 10469. 34254782 10.1021/acs.chemrev.1c00241

[3] V. L. Dao Thi , K. Herbst , K. Boerner , M. Meurer , L. P. Kremer , D. Kirrmaier , A. Freistaedter , D. Papagiannidis , C. Galmozzi , M. L. Stanifer , S. Boulant , S. Klein , P. Chlanda , D. Khalid , I. Barreto Miranda , P. Schnitzler , H.‐G. Kräusslich , M. Knop , S. Anders , Sci. Transl. Med. 2020, 12, 7075. 10.1126/scitranslmed.abc7075 PMC7574920 32719001

[4] L. Xu , J. Duan , J. Chen , S. Ding , W. Cheng , Anal. Chim. Acta. 2021, 1148, 238187. 33516384 10.1016/j.aca.2020.12.062

[5] M. S. Reid , X. C. Le , H. Zhang , Angew. Chem., Int. Ed. 2018, 57, 11856. 10.1002/anie.201712217 29704305

[6] C. Shi , Q. Liu , C. Ma , W. Zhong , Anal. Chem. 2014, 86, 336. 24345199 10.1021/ac4038043

[7] T. D. Schmittgen , B. A. Zakrajsek , A. G. Mills , V. Gorn , M. J. Singer , M. W. Reed , Anal. Biochem. 2000, 285, 194. 11017702 10.1006/abio.2000.4753

[8] J. V. J. Silva Júnior , I. Merchioratto , P. S. B. de Oliveira , T. R. Rocha Lopes , P. C. Brites , E. M. de Oliveira , R. Weiblen , E. F. Flores , J. Virol. Methods. 2021, 288, 114007. 33130151 10.1016/j.jviromet.2020.114007 PMC7598561

[9] Q. Yao , Y. Wang , J. Wang , S. Chen , H. Liu , Z. Jiang , X. Zhang , S.‐M. Liu , Q. Yuan , X. Zhou , ACS Nano. 2018, 12, 6777. 29924598 10.1021/acsnano.8b01950

[10] C. Li , H. Chen , J. Hu , C. Zhang , Chem. Sci. 2020, 11, 5724. 32864084 10.1039/d0sc01652g PMC7433776

[11] A. Joneja , X. Huang , Anal. Biochem. 2011, 414, 58. 21342654 10.1016/j.ab.2011.02.025 PMC3108800

[12] K. Montagne , R. Plasson , Y. Sakai , T. Fujii , Y. Rondelez , Mol. Syst. Biol. 2011, 7, 466. 21283142 10.1038/msb.2010.120 PMC3063689

[13] A. Baccouche , K. Montagne , A. Padirac , T. Fujii , Y. Rondelez , Methods. 2014, 67, 234. 24495737 10.1016/j.ymeth.2014.01.015

[14] K. Montagne , G. Gines , T. Fujii , Y. Rondelez , Nat. Commun. 2016, 7, 13474. 27845324 10.1038/ncomms13474 PMC5116077

[15] L. Qian , E. Winfree , Science. 2011, 332, 1196. 21636773 10.1126/science.1200520

[16] F. Wang , H. Lv , Q. Li , J. Li , X. Zhang , J. Shi , L. Wang , C. Fan , Nat. Commun. 2020, 11, 1. 31913309 10.1038/s41467-019-13980-y PMC6949259

[17] C. Kieffer , A. J. Genot , Y. Rondelez , G. Gines , Advanced Biology. 2023, 7, 2200203. 10.1002/adbi.202200203 36709492

[18] Y. Rondelez , G. Gines , ACS Sens. 2020, 5, 2430. 32602335 10.1021/acssensors.0c00593 PMC7460561

[19] J. Wang , J. Chen , S. Sen , J. Cell. Physiol. 2016, 231, 25. 26031493 10.1002/jcp.25056 PMC8776330

[20] G. Gines , R. Menezes , K. Nara , A.‐S. Kirstetter , V. Taly , Y. Rondelez , Sci. Adv. 2020, 6, 5952. 10.1126/sciadv.aay5952 PMC6976291 32010788

[21] P. Biswal , A. Lalruatfela , S. K. Behera , S. Biswal , B. Mallick , IUBMB Life. 2024, 76, 108. 37792370 10.1002/iub.2786

[22] J. Balzeau , M. R. Menezes , S. Cao , J. P. Hagan , Frontiers in Genetics. 2017, 8. 10.3389/fgene.2017.00031 PMC5368188 28400788

[23] G. Gines , R. Menezes , W. Xiao , Y. Rondelez , V. Taly , Molecular Aspects of Medicine. 2019, 72, 100832. 31767382 10.1016/j.mam.2019.11.002

[24] S. Gong , S. Zhang , F. Lu , W. Pan , N. Li , B. Tang , Anal. Chem. 2021, 93, 11899. 34427091 10.1021/acs.analchem.1c02533

[25] Q. A. Phan , L. B. Truong , D. Medina‐Cruz , C. Dincer , E. Mostafavi , Biosens. Bioelectron. 2022, 197, 113732. 34741959 10.1016/j.bios.2021.113732

[26] M. M. Kaminski , O. O. Abudayyeh , J. S. Gootenberg , F. Zhang , J. J. Collins , Nat. Biomed. Eng. 2021, 5, 643. 34272525 10.1038/s41551-021-00760-7

[27] J. S. Gootenberg , O. O. Abudayyeh , J. W. Lee , P. Essletzbichler , A. J. Dy , J. Joung , V. Verdine , N. Donghia , N. M. Daringer , C. A. Freije , C. Myhrvold , R. P. Bhattacharyya , J. Livny , A. Regev , E. V. Koonin , D. T. Hung , P. C. Sabeti , J. J. Collins , F. Zhang , Science. 2017, 356, 438. 28408723 10.1126/science.aam9321 PMC5526198

[28] C. Myhrvold , C. A. Freije , J. S. Gootenberg , O. O. Abudayyeh , H. C. Metsky , A. F. Durbin , M. J. Kellner , A. L. Tan , L. M. Paul , L. A. Parham , K. F. Garcia , K. G. Barnes , B. Chak , A. Mondini , M. L. Nogueira , S. Isern , S. F. Michael , I. Lorenzana , N. L. Yozwiak , B. L. MacInnis , I. Bosch , L. Gehrke , F. Zhang , P. C. Sabeti , Science. 2018, 360, 444. 29700266 10.1126/science.aas8836 PMC6197056

[29] J. S. Chen , E. Ma , L. B. Harrington , M. Da Costa , X. Tian , J. M. Palefsky , J. A. Doudna , Science. 2018, 360, 436. 29449511 10.1126/science.aar6245 PMC6628903

[30] H. Tas , L. Grozinger , R. Stoof , V. de Lorenzo , Á. Goñi‐Moreno , Nat. Commun. 2021, 12, 355. 33441561 10.1038/s41467-020-20656-5 PMC7806840

[31] J. A. N. Brophy , C. A. Voigt , Nat. Methods. 2014, 11, 508. 24781324 10.1038/nmeth.2926 PMC4230274

[32] S. Okumura , G. Gines , N. Lobato‐Dauzier , A. Baccouche , R. Deteix , T. Fujii , Y. Rondelez , A. J. Genot , Nature. 2022, 610, 496. 36261553 10.1038/s41586-022-05218-7

[33] K. M. Cherry , L. Qian , Nature. 2018, 559, 370. 29973727 10.1038/s41586-018-0289-6

[34] R. Lopez , R. Wang , G. Seelig , Nat. Chem. 2018, 10, 746. 29713032 10.1038/s41557-018-0056-1

[35] C. Zhang , Y. Zhao , X. Xu , R. Xu , H. Li , X. Teng , Y. Du , Y. Miao , H. Lin , D. Han , Nat. Nanotechnol. 2020, 15, 709. 32451504 10.1038/s41565-020-0699-0

[36] R. Daniel , J. R. Rubens , R. Sarpeshkar , T. K. Lu , Nature. 2013, 497, 619. 23676681 10.1038/nature12148

[37] J. A. Sanchez , K. E. Pierce , J. E. Rice , L. J. Wangh , Proc. Natl. Acad. Sci. USA. 2004, 101, 1933. 14769930 10.1073/pnas.0305476101 PMC357030

[38] L. D. Smith , S. Nalla , C.‐W. Kuo , M. Kohli , A. M. Smith , Analyst. 2022, 147, 2936. 35695478 10.1039/d2an00263a PMC11247439

[39] V. K. Nair , C. Sharma , S. Ghosh , ChemRxiv 2022, 10.26434/chemrxiv-2022-mxtb3-v3.

[40] A. Yamagata , R. Masui , Y. Kakuta , S. Kuramitsu , K. Fukuyama , Nucleic Acids Res. 2001, 29, 4617. 11713311 10.1093/nar/29.22.4617 PMC92510

[41] Y. Rondelez , G. Gines , in MicroRNA Detection and Target Identification: Methods and Protocols, (Ed.: T. Dalmay ), Springer US, New York, NY 2023, pp. 89–102.

