# Supplementary material for: Coupling Exponential to Linear Amplification for Endpoint Quantitative Analysis
Source: Adv Sci (Weinh). 2024 Apr 9;11(21):2309386. doi: 10.1002/advs.202309386 (PMC11151046; doi:10.1002/advs.202309386)
Supplement: Supplementary file 1 — Supporting Information [file ADVS-11-2309386-s001.pdf]

ADVANCED SCIENCE
Open Access

Supporting Information

for *Adv. Sci.*, DOI 10.1002/advs.202309386

Coupling Exponential to Linear Amplification for Endpoint Quantitative Analysis

*Coline Kieffer, Yannick Rondelez and Guillaume Gines**

Supporting Information

Coupling Exponential to Linear Amplification for Endpoint Quantitative Analysis

*Coline Kieffer, Yannick Rondelez, Guillaume Gines**

Table of Contents

Figure S1. Detailed chemical reaction network of the linear amplification module. 2
Figure S2. Detailed chemical reactions of the CELIA network. 2
Figure S3. Detailed chemical reactions of the CELIA network for microRNA detection 3
Figure S4. Detailed chemical reactions of the CELIA network embedding a signal inverter function. 3
Figure S5. Detailed chemical reactions of the linear amplification circuit for let-7a detection 4
Figure S6. Extended data from Figure 3. 5
Figure S7. Linear signal normalization. 6
Figure S8. Tunability of the linear amplification module in the inverter function. 7
Figure S9. Inhibition of ω production by the killer template αkβ. 8
Figure S10. Effect of the killer template concentration on the inhibition of ω production. 9
Figure S11. Extended data from Figure 5. 10
Figure S12. Extended data from the Figure 4C. 11
Figure S13. Strand displacement linear amplification for miRNA detection. 11
Figure S14. RT-qPCR miRNA calibration curves. 12
Figure S15. Extended data from the Figure 4D. 13
Figure S16. Analysis of synthetic samples containing let-7a and miR-203a by RT-qPCR. 14
Figure S17. Example of template design. 15
Figure S18. Experimental conditions used in this study. 16

Table S1. Nucleic acid sequences used throughout this study. 17

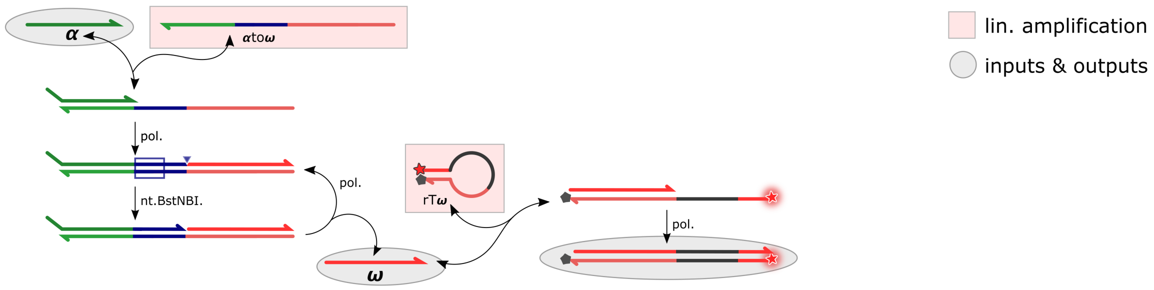

Figure S1. Detailed chemical reaction network of the linear amplification module (Figure 2).

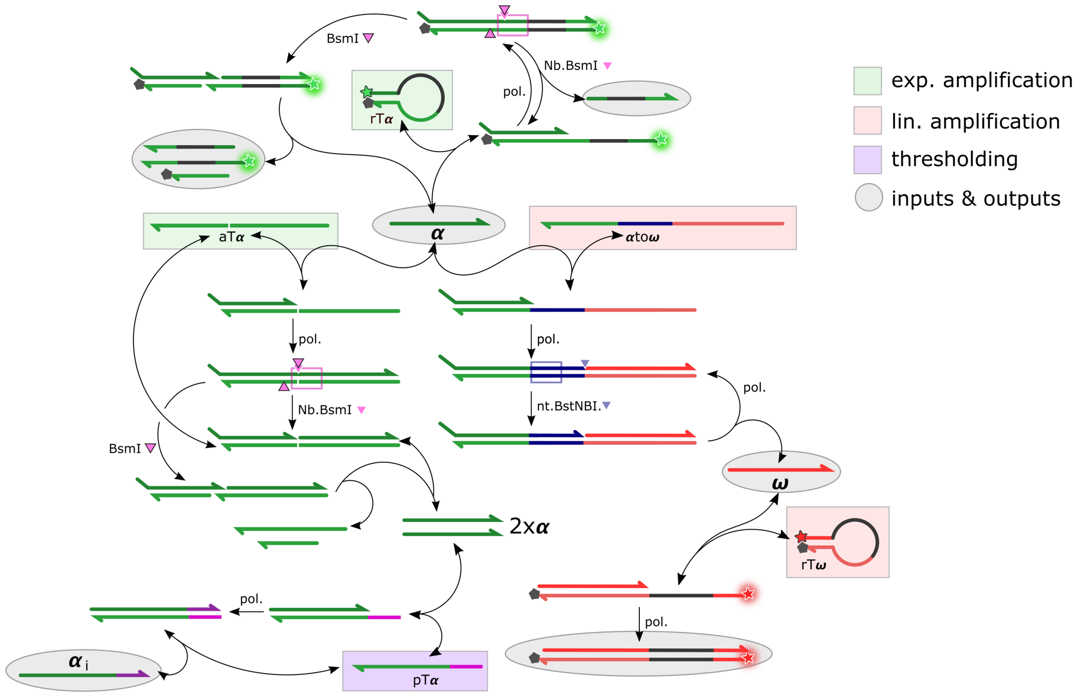

Figure S2. Detailed chemical reactions of the CELIA network (Figure 3).

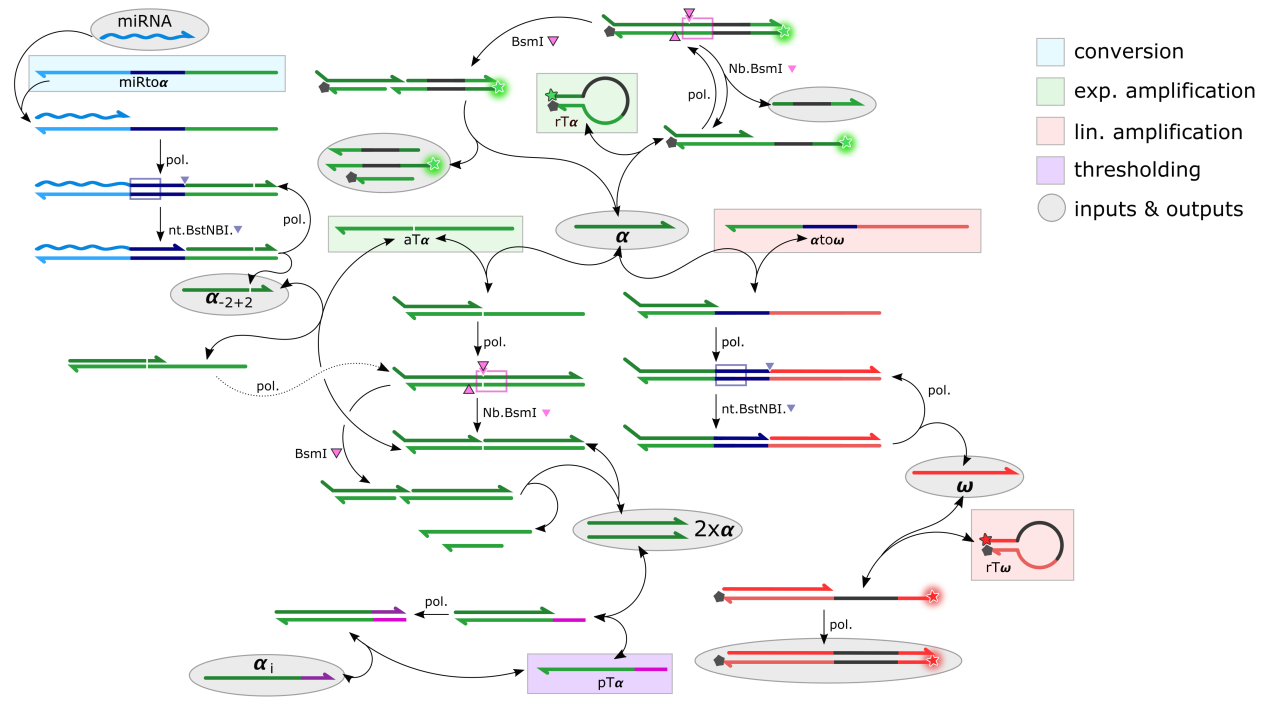

Figure S3. Detailed chemical reactions of the CELIA network for microRNA detection (Figure 5). The dotted arrow indicates that reaction intermediates are not represented for the sake of clarity.

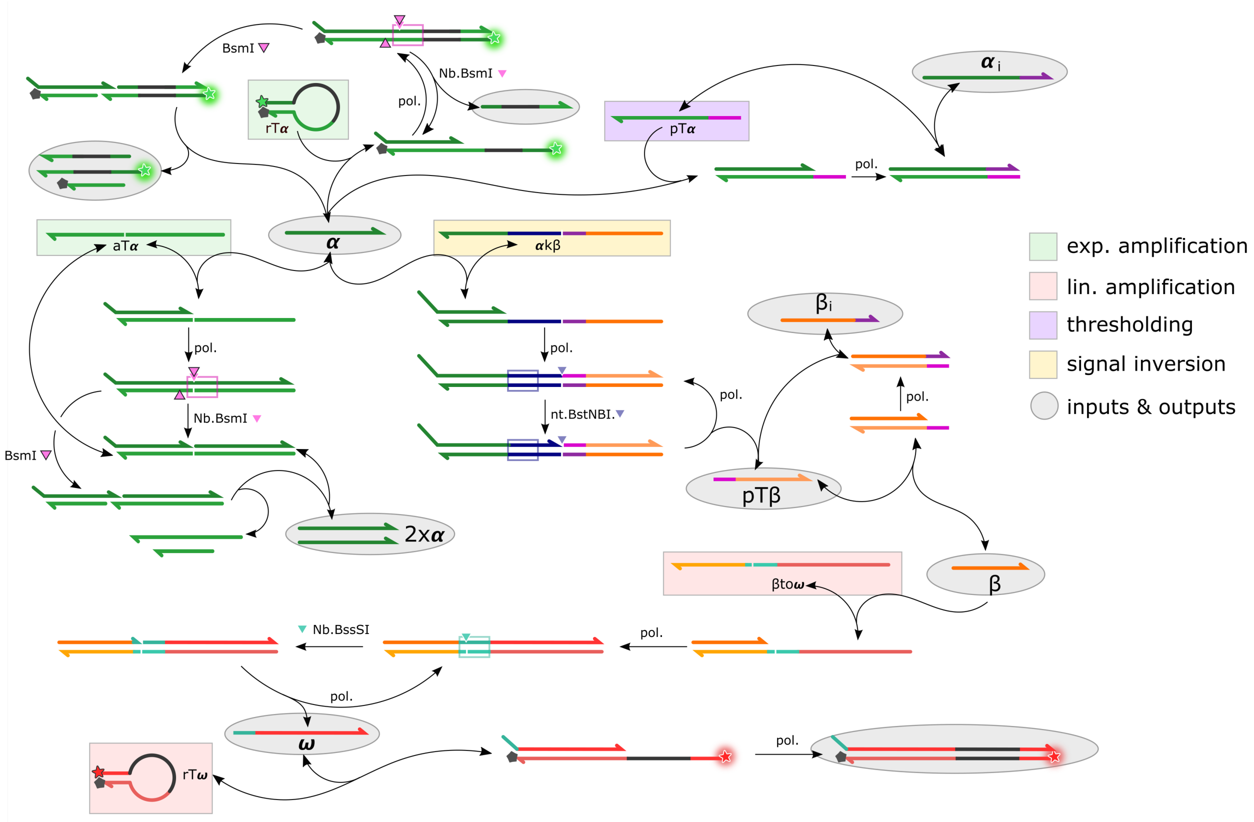

Figure S4. Detailed chemical reactions of the CELIA network embedding a signal inverter function (Figure 4).

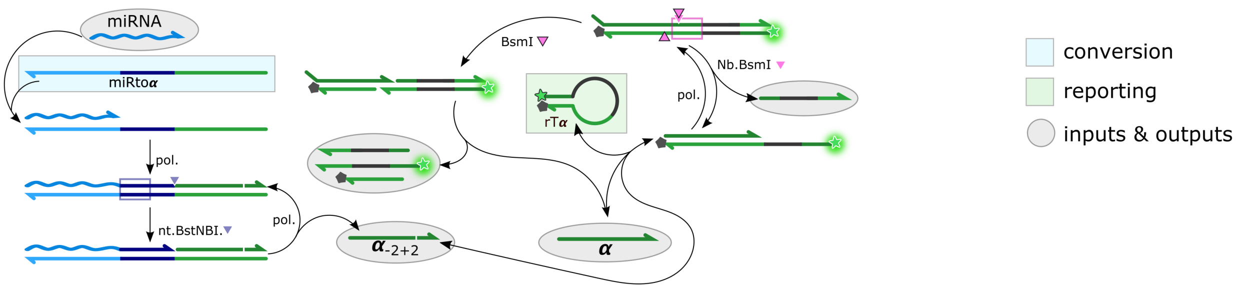

Figure S5. Detailed chemical reactions of the linear amplification circuit for let-7a detection (cf. Figure S13).

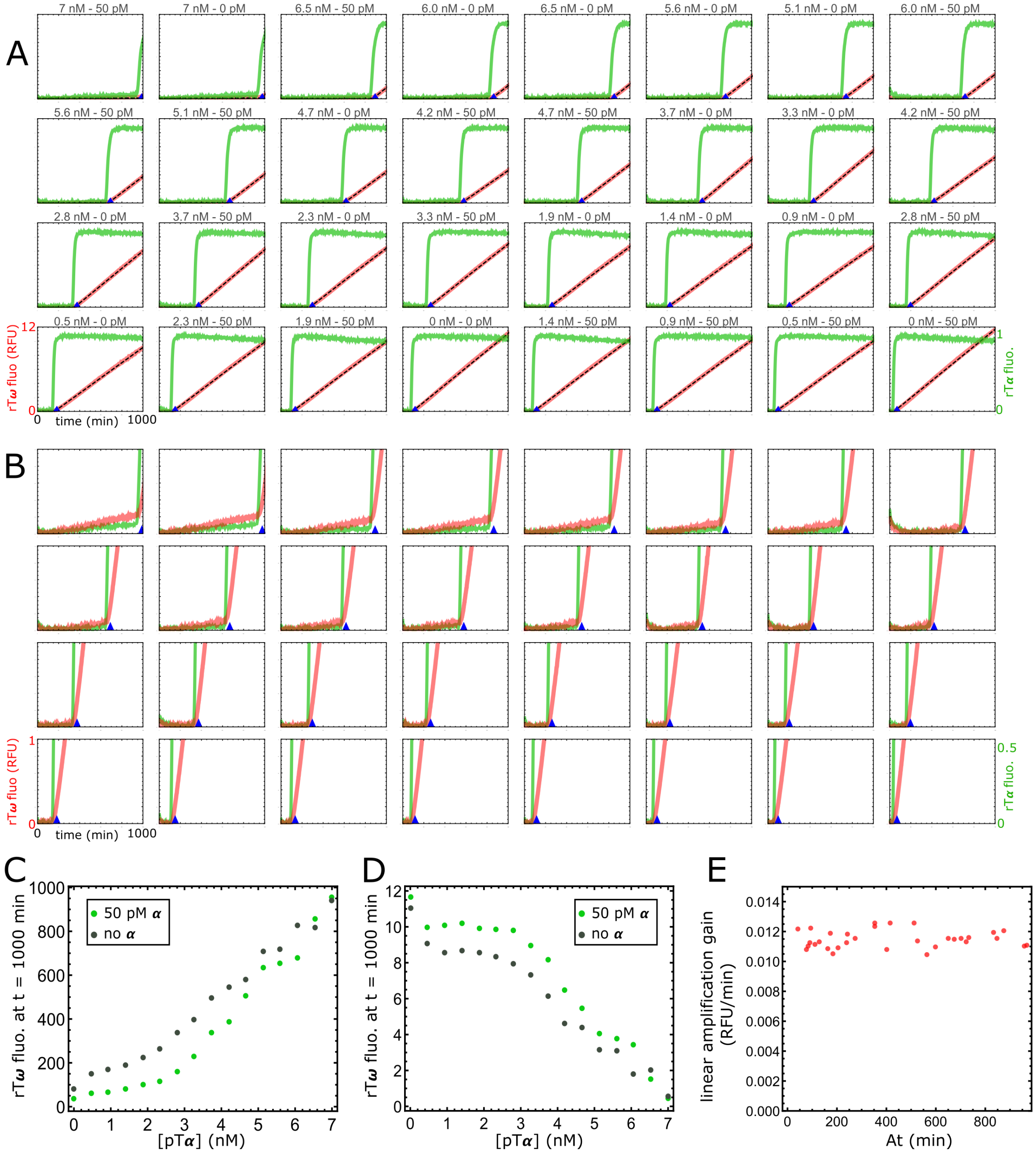

Figure S6. Extended data from Figure 3. (A) Amplification curves for individual samples spiked with various concentrations of pTα and α. The linear amplification time trace (red curve) is fitted with a piecewise function (black dashed curve) $f(x) = \{b, if\ x \leq At; a.x + b - a.At, if\ x > At$, where b is the baseline constant, At is the amplification time and a is the linear amplification gain. The corresponding exponential α amplification curve is represented as the gray curve. On the top on each panel are indicated the concentration of pTα (from 0 to 7 nM) and α (0 or 50 pM) (B) Zoom on early emergence of the rTω fluorescence demonstrates the sharp transition from a near-null to a constant ω production, concomitantly to the detection of the exponential amplification. (C) Amplification time as a function of [pTα]. (D) Endpoint (1000 minutes) fluorescence of rTω as a function of [pTα]. (E) The linear amplification gain extracted from the fit is plotted as a function of the amplification time. The gain is constant (CV ~5%) and shows no correlation with the amplification time, demonstrating the persistence of the DNA-enzyme reaction network toward long incubation time at 50 °C.

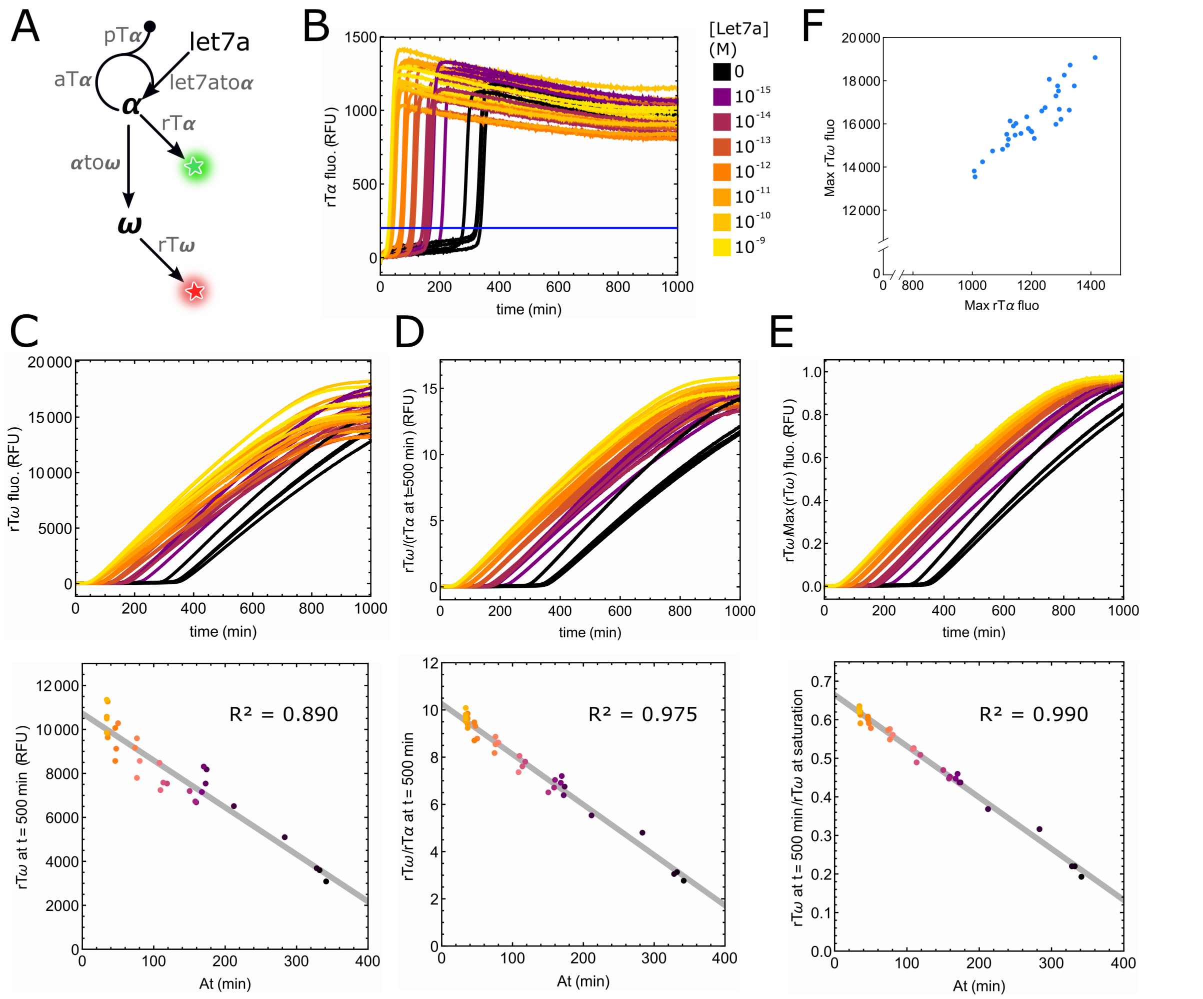

Figure S7. Linear signal normalization. The let-7a detection circuit is spiked with various concentration of let-7a, resulting in the modulation of the α amplification time. (B) Time traces of the exponential amplification (rTα fluorescence). The blue line depicts the signal threshold for extracting the amplification time (At). (C) top: Non-normalized time traces of the linear amplification (rTω). bottom: endpoint signal (t = 500 min) as a function of At. (D) top: Time traces of the linear amplification (rTω) normalized with respect to the endpoint fluorescence of rTα (t=500 min). bottom: normalized endpoint ω signal (t = 500 min) as a function of At. (E) top: time traces of the linear amplification (rTω) normalized with respect to the ω signal at saturation (~1000 minutes). bottom: normalized endpoint ω signal (t = 500 min) as a function of At. (F) Maximum fluorescence at saturation of rTω versus rTα. Unnormalized endpoint linear signal (C) show a substantial deviation from the expected proportionality with At (R^2 = 0.89). The proportionality is almost perfect when dividing rTω signal by its signal at saturation for the same well (E). However, such normalization is not possible in a standard endpoint experiment, as the saturation is reached after the experiment is stopped (here the endpoint time is 500 minutes while the saturation is reached in about 1000 minutes). Alternatively, the rTω signal can be normalized by the rTα signal for the same endpoint time (D), which significantly improves the correlation with At. This indicates that most of the observed dispersion results from instrumental noise, more specifically inter-well variations of the optical signal, which applies to both rTα and rTω (F).

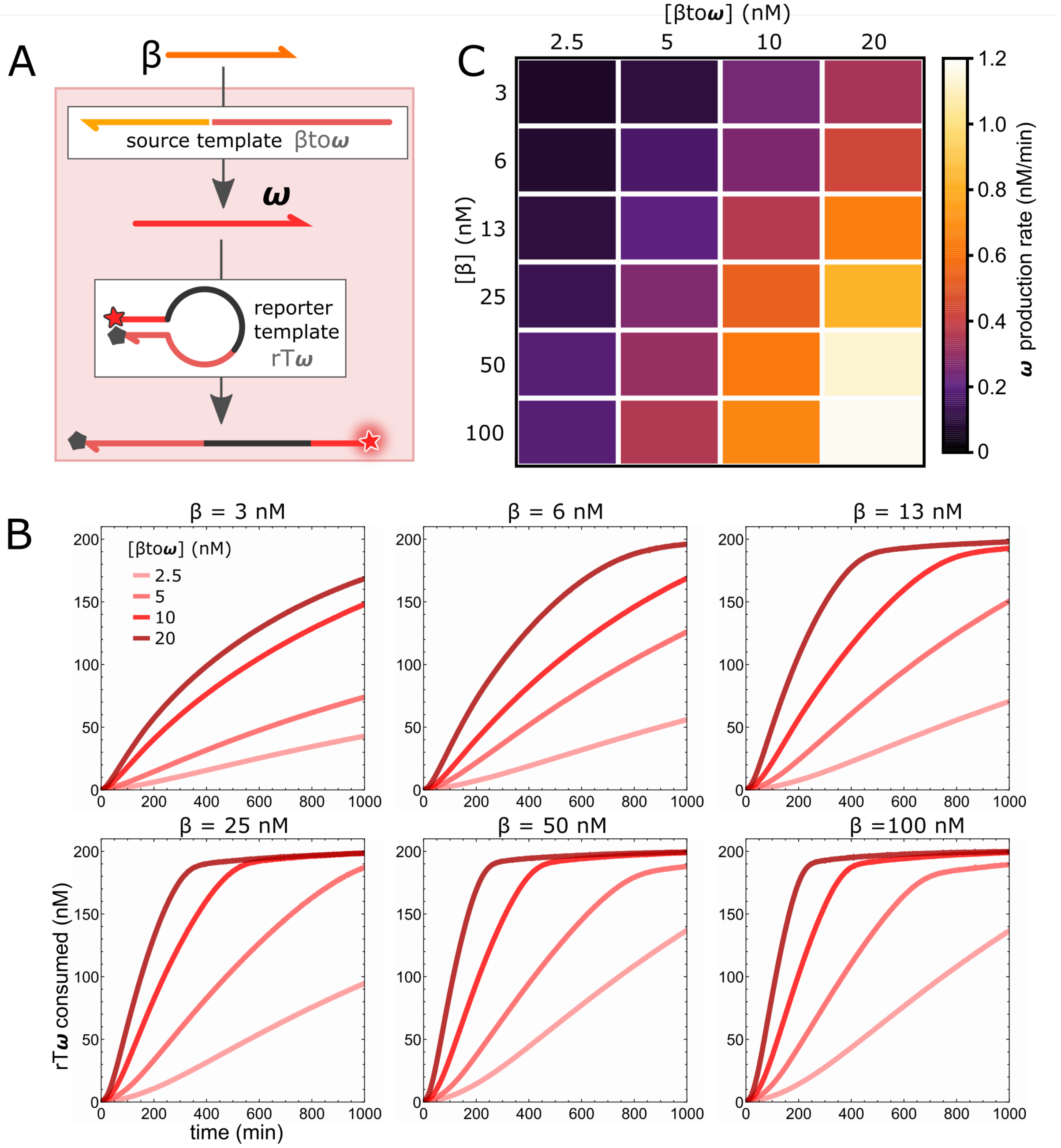

Figure S8. Tunability of the linear amplification module in the inverter function. (A) Architecture of the linear amplification module. (B) Real-time monitoring of the linear amplification module for various concentrations of βtoω and β. (C) Array plot of the production rate of ω. The more βtoω converter template or β input, the faster the production of ω.

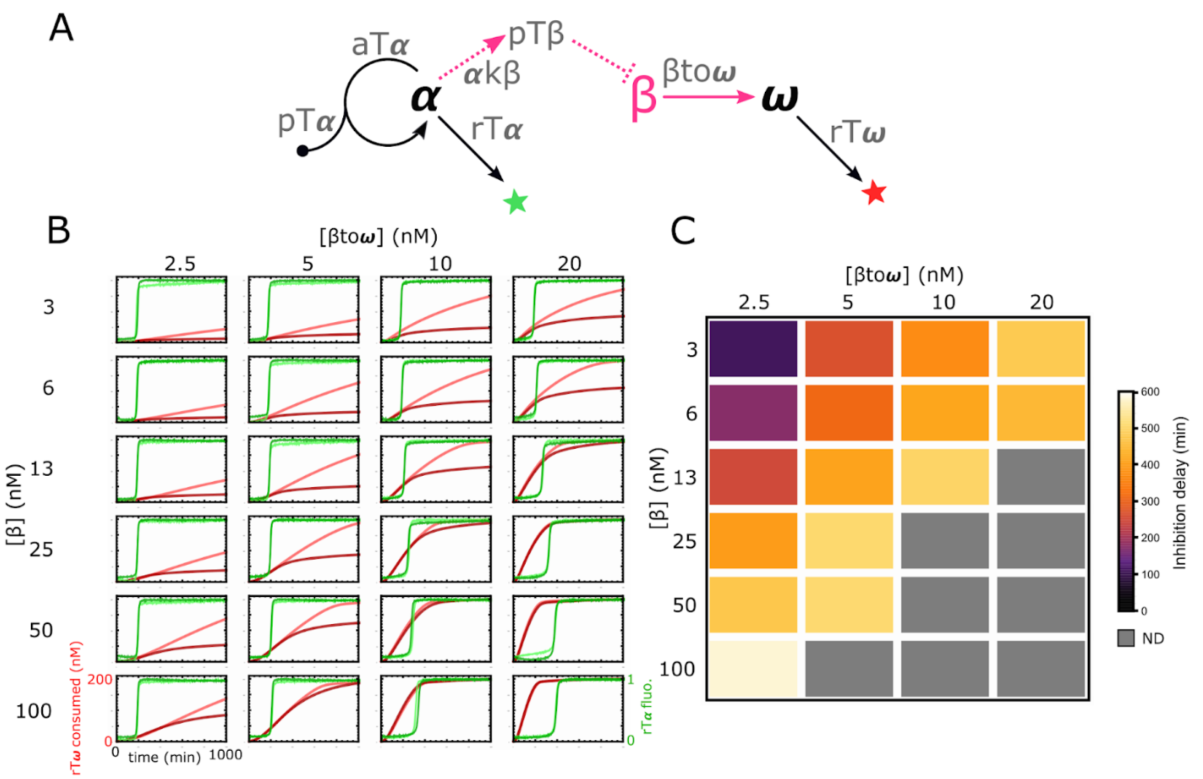

Figure S9. Inhibition of ω production by the killer template αkβ. (A) The inverter circuit, in absence or presence of αkβ (5 nM) is run for various concentrations of βtoω and β. (B) Time traces of the exponential (green) and linear (red) amplification reactions in absence (light color) and presence (dark color) of αkβ. (C) Array plot of the inhibition delay, which correspond to difference between the amplification time and the time the linear amplification rate goes below 10 pM of consumed rTω per minute. The more βtoω or β input, the longer it takes for the killer template to reach the quasi-complete inhibition of ω production. For high concentrations of βtoω and β (gray boxes), rTω is entirely consumed before the inhibition reaction crosses the 10 pM/min threshold, which prevents from determining the inhibition delay.

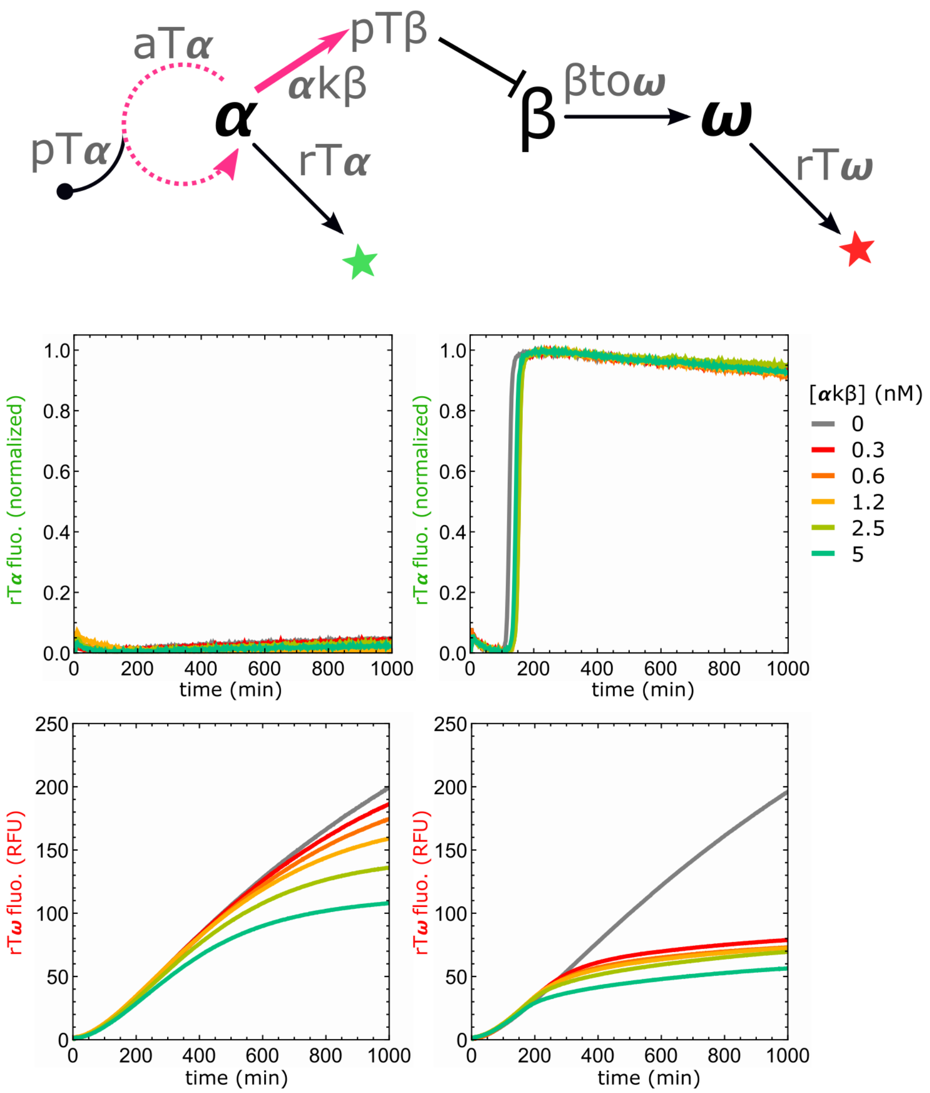

Figure S10. Effect of the killer template concentration on the inhibition of ω production. (A) The inverter circuit in the absence (to test the kT leakage) and presence (to test the kT inhibition efficiency) of aTα is run in the presence of a varying concentration of αkβ. (B) Time traces of the exponential (top) and linear (bottom) amplification reaction in the absence (left) or presence (right) of aTα. In the absence of aTα, we noticed that the linear amplification rate decreases over time and this effect is all the more important as αkβ concentration is increased. This suggests that αkβ can slowly self-activate and that this leaky reaction gradually consumes the pool of β, eventually reducing the ω production rate. When loaded by aTα, the inhibition is substantially more efficient than the self-activation process. As expected, the higher [αkβ], the lower the inhibition delay following α exponential amplification.

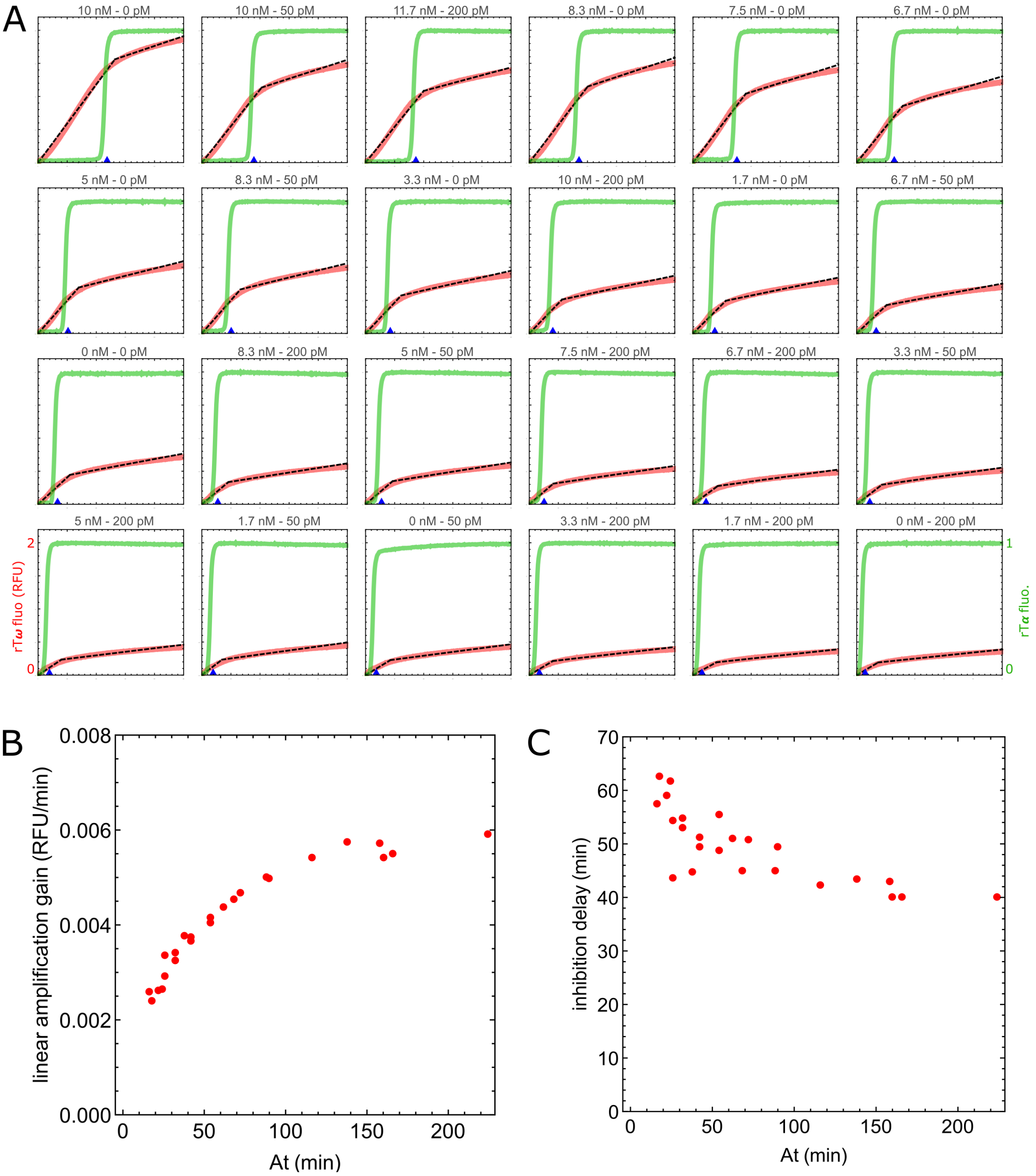

Figure S11. Extended data from Figure 5. (A) Amplification curves for individual samples spiked with various concentrations of pTα and α. The linear amplification time trace (red curve) is fitted with a piecewise function (black dashed curve) $f(x) = \{a.x, if\ x \leq At + d; a.(At + d) + a'.(x + At + d), if\ x > At + d$, where At is the amplification time extracted from the exponential amplification curve (represented as a blue triangle), d is the inhibition delay, a and a' are initial linear amplification gain and the residual linear rate, respectively. The corresponding exponential α amplification curve is superimposed as the green curve. On the top on each panel are indicated the concentration of pTα (from 0 to 10 nM) and α (0, 50 or 200 pM). (B) Linear amplification gain (extracted from the fit) as a function of At. We observe that for short At, this value is correlated to the gain. This is explained by acceleration of the ω production in the early phase of the linear amplification (cf. also Figure S10), which introduces a

bias in the linear fit. (C) Inhibition delay (d in the fitting function) versus At. Similarly, this value is – negatively – correlated to At due to uncertainties in the fitting function.

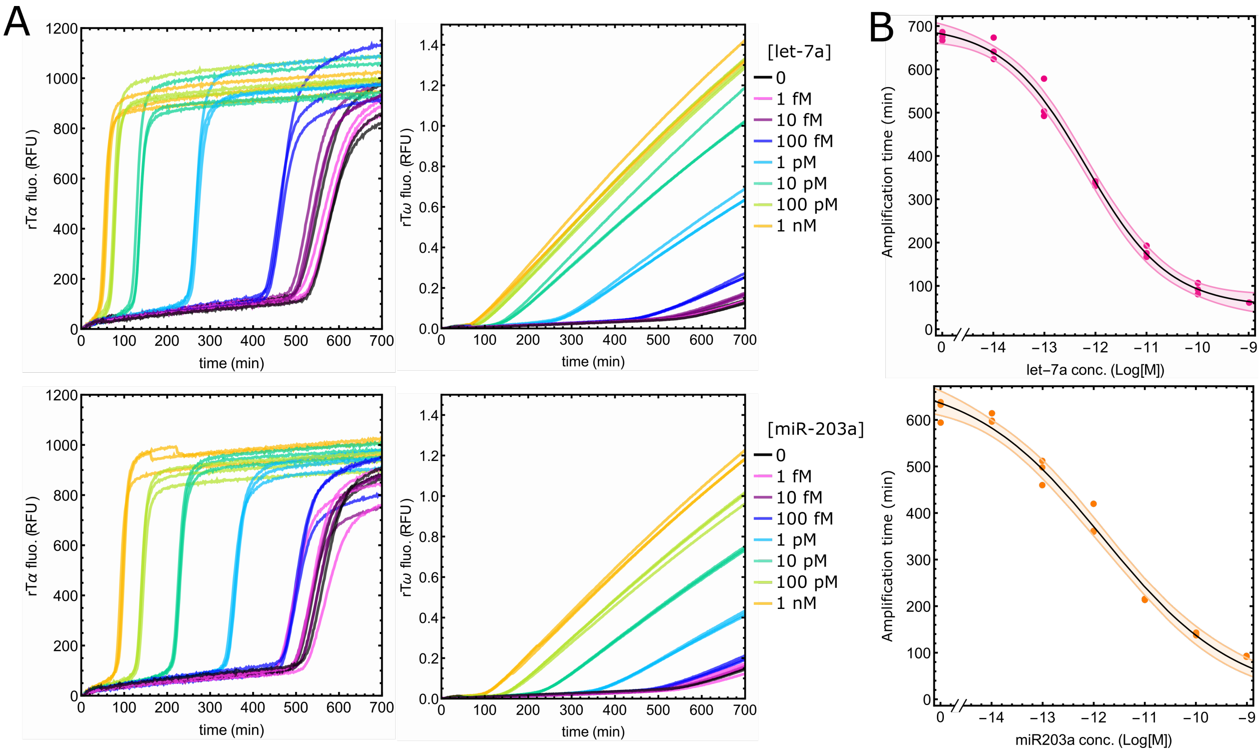

Figure S12. Extended data from the Figure 4C. (A) Exponential (left) and linear (right) amplification time traces for let-7a (top) and miR-203a (bottom) calibration curves. (B) Amplification time calibration curves for let-7a (top) and miR-203a (bottom). The data points were fitted with a sigmoid function $f(x) = \frac{a}{1+e^{-k(x-b)}} + c$, where k is the steepness of the curve, b denotes the value of x at the sigmoid midpoint and c and a+c represent the lower and higher asymptotes, respectively. The colored shaded area represents the 95 % confidence interval on the fit parameters.

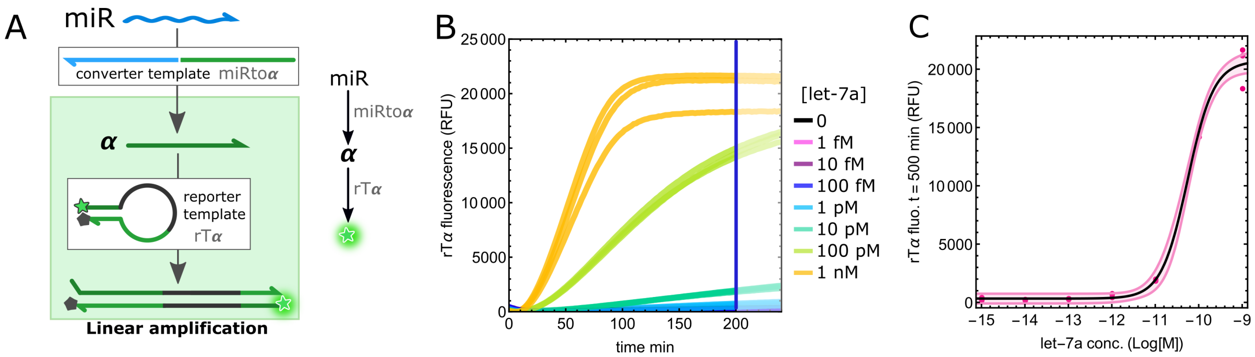

Figure S13. Strand displacement linear amplification for miRNA detection. (A) The linear amplification is achieved here by omitting the exponential amplification module and by directly connecting the output of the miRtoα converter template to the reporter template rTα. (B) Time traces of the linear amplification of a triplicate experiment for various

concentration of the target let-7a. (C) Calibration curve of the endpoint rTα fluorescence (t = 200 min) as a function of let-7a concentration.

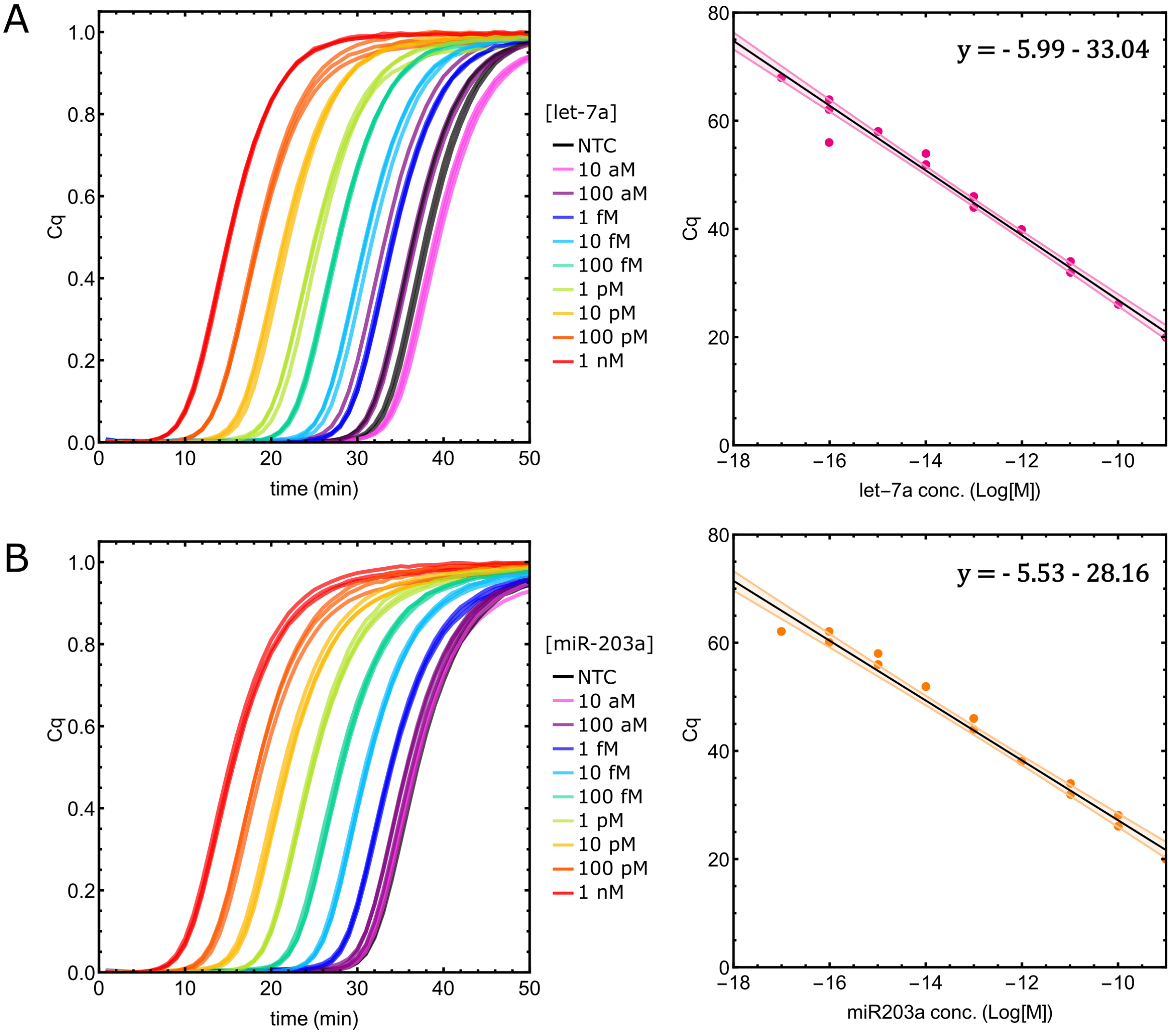

Figure S14. RT-qPCR miRNA calibration curves. (A) for let-7a and (B) for miR-203. The left panels represent qPCR amplification curves for a technical triplicate experiment. The right panels show the extracted calibration curves. All data points (except the no template control (NTC) were fitted with a linear regression (black line). The colored shaded area represents the 95 % confidence interval on the fit parameters.

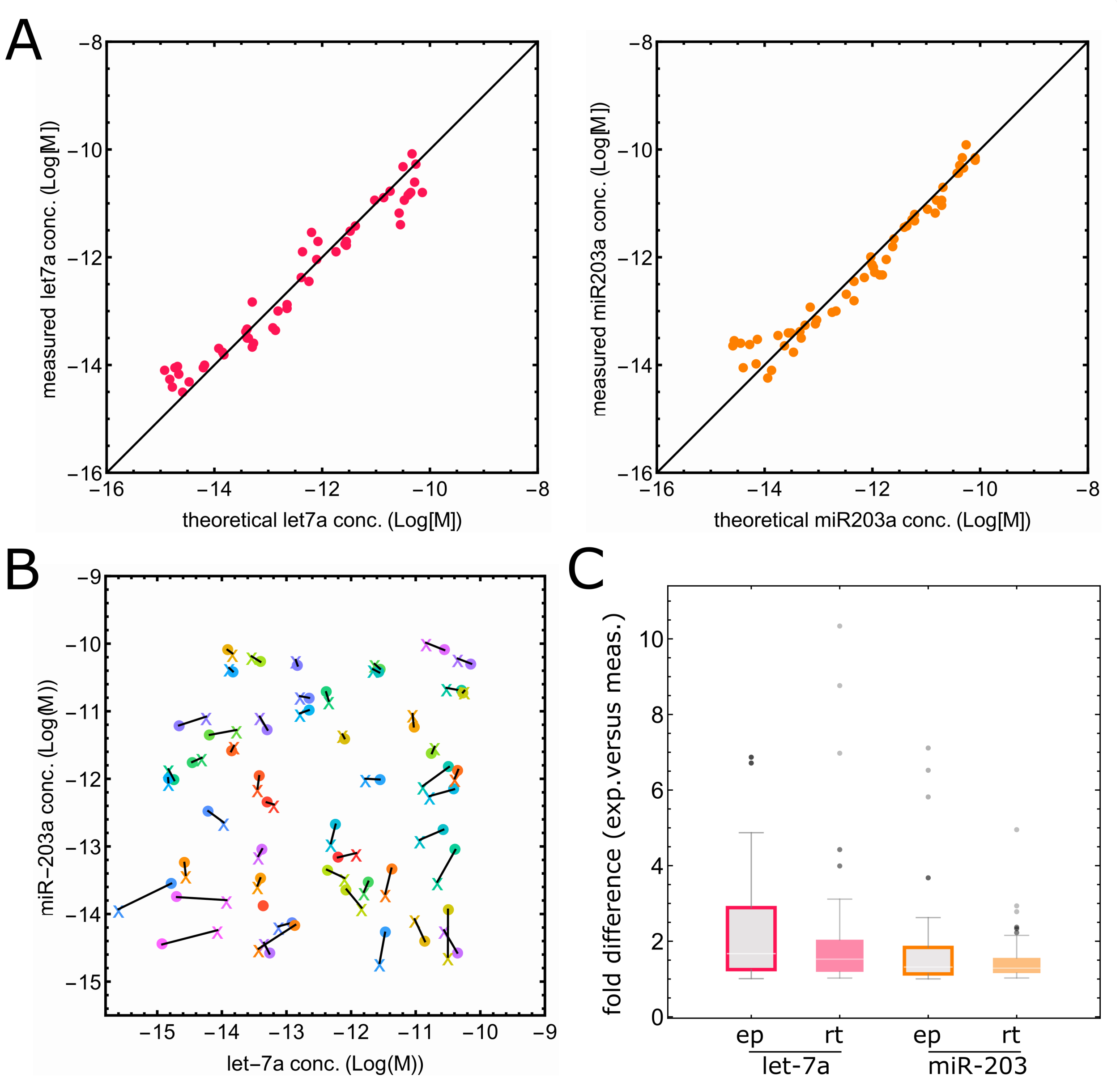

Figure S15. Extended data from the Figure 4D. (A) Expected concentration versus concentration measured from the endpoint rTω fluorescence for let-7a (left) and miR-203a (right). (B) 2D pattern of expected concentration (disks) versus concentration measured from the real-time amplification time traces (crosses). (C) Fold differences distribution computed for the endpoint (ep) or real-time (rt) readout.

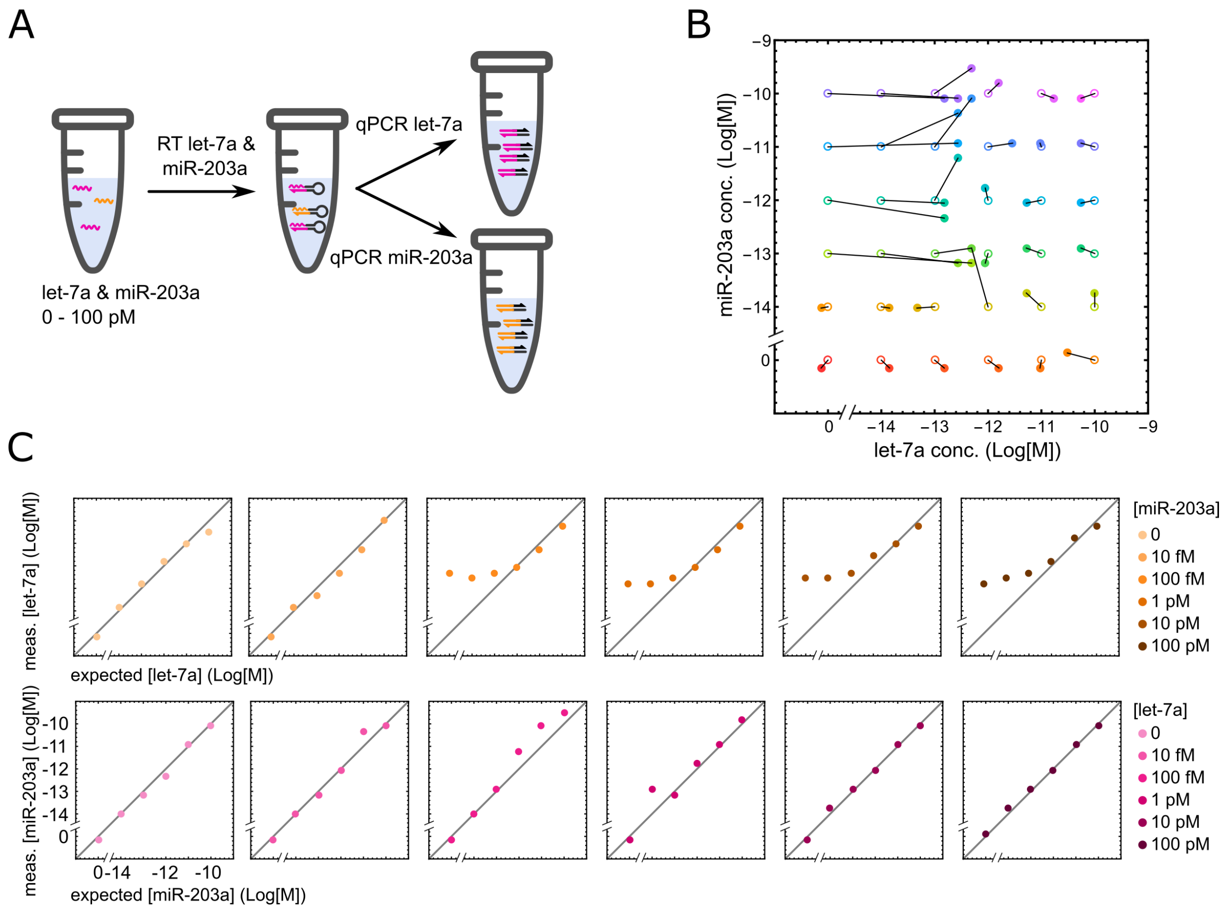

Figure S16. Analysis of synthetic samples containing let-7a and miR-203a by RT-qPCR. (A) 36 samples were prepared containing both miRNA, each at a concentration comprised between 0 and 100 pM. The reverse transcription step was performed in one pot using the two stem-loop primers (cf. Experimental section). The qPCR quantification of the resulting cDNA was performed separately for each target using the corresponding set of primers and probe. (B) Quantification results for all samples (plain disk = expected concentration, circles = measured concentration). (C) Measured concentration plotted as a function of the expected concentration for let-7a (top) and miR-203 (bottom), from left to right as the concentration of the other miRNA increases. It appears that the concentration of let-7a was largely overestimated in samples with low level of let-7a and high concentration of miR-203a, indicating a significant interference of miR-203a on the let-7a assay.

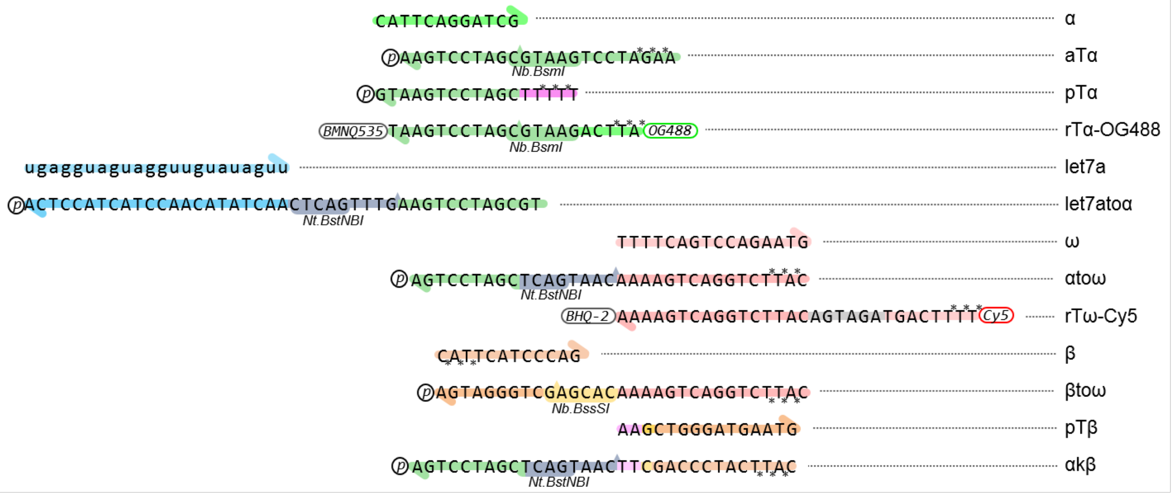

Figure S17. Example of template design. "*" are phosphorothioate bonds for 5'end protection. "P" indicates 3' phosphate modification. Small protrusion indicates the nick site on the opposite strand.

Figure 2	
α	20 nM
αtoω	↗
rTω-Cy5	↗
BSA	200 µg/mL
Vent(exo-)	70 u/mL
Nt.BstNBI	10 u/mL
temperature	50 °C

Figure 3 – Figure S6	
αbis	0-50 pM
aTαbis	50 nM
pTαbis	↗
rTαbis-Atto488	20 nM
αbistoω	2 nM
rTω-Cy5	200 nM
BSA	200 µg/mL
Vent(exo-)	70 u/mL
Nt.BstNBI	30 u/mL
Nb.BsmI	200 u/mL
BsmI	70 u/mL
ttRecJ	23 nM
temperature	48 °C

Figure 5 – Figure S11	
α	0-50-500 pM
aTα	50 nM
pTα	↗
rTα-TET	20 nM
αkβ	2 nM
β	20 nM
βtoω	6 nM
rTω-Cy5	200 nM
BSA	200 µg/mL
Vent(exo-)	70 u/mL
Nb.BssSI	40 u/mL
Nt.BstNBI	25 u/mL
Nb.BsmI	200 u/mL
BsmI	50 u/mL
ttRecJ	3 nM
dNTPs	125 µM
temperature	46 °C

Figure 4 – Figure S12-15	
let7atoα or miR203atoα	0.5 nM
aTα	50 nM
pTα	8 nM
rTα-OG488	25 nM
αtoω	5 nM
rTω-Cy5	250 nM
let-7a and/or miR-203a	↗
BSA	200 µg/mL
Vent(exo-)	80 u/mL
Nt.BstNBI	30 u/mL
Nb.BsmI	200 u/mL
BsmI	100 u/mL
ttRecJ	13 nM
dNTPs	25 µM
temperature	50 °C

Figure 6	
let-7a	↗
RCα	1 nM
RCω	1 nM
MBω-Cy5	200 nM
BSA	600 µg/mL
Φ29 DNA polymerase	100 u/mL
Nb.BbvCI	200 u/mL
dNTPs	1 mM
SYBR Gold	1.25 X
temperature	37 °C

Figure S7	
let7atoα	0.5 nM
aTα	50 nM
pTα	8 nM
rTα-OG488	25 nM
αtoω	5 nM
rTω-Cy5	150 nM
let-7a	↗
BSA	200 µg/mL
Vent(exo-)	80 u/mL
Nt.BstNBI	30 u/mL
Nb.BsmI	200 u/mL
BsmI	100 u/mL
ttRecJ	13 nM
dNTPs	25 µM
temperature	50 °C

Figure S8	
aTα	50 nM
pTα	6 nM
rTα-OG	20 nM
β	↗
βtoω	↗
rTω-Cy5	200 nM
BSA	200 µg/mL
Vent(exo-)	70 u/mL
Nb.BssSI	40 u/mL
Nt.BstNBI	30 u/mL
Nb.BsmI	200 u/mL
BsmI	50 u/mL
ttRecJ	3 nM
dNTPs	125 µM
temperature	48 °C

Figure S9	
aTα	50 nM
pTα	6 nM
rTα-OG	20 nM
αkβ	0 or 5 nM
β	↗
βtoω	↗
rTω-Cy5	200 nM
BSA	200 µg/mL
Vent(exo-)	70 u/mL
Nb.BssSI	40 u/mL
Nt.BstNBI	30 u/mL
Nb.BsmI	200 u/mL
BsmI	50 u/mL
ttRecJ	3 nM
dNTPs	125 µM
temperature	48 °C

Figure S10	
aTα	50 nM
pTα	5 nM
rTα-OG	20 nM
αkβ	↗
β	70 nM
βtoω	5 nM
rTω-Cy5	200 nM
BSA	200 µg/mL
Vent(exo-)	70 u/mL
Nb.BssSI	40 u/mL
Nt.BstNBI	30 u/mL
Nb.BsmI	200 u/mL
BsmI	50 u/mL
ttRecJ	3 nM
dNTPs	125 µM
temperature	48 °C

Figure S13	
let7atoα	5 nM
rTα-OG488	150 nM
let-7a	↗
BSA	200 µg/mL
Vent(exo-)	70 u/mL
Nt.BstNBI	20 u/mL
BsmI	200 u/mL
temperature	50 °C

Figure S18. Experimental conditions used in this study.

Table S1. Nucleic acid sequences used throughout this study. "*" and "p" denote phosphorothioate backbone modification and 3' phosphate moiety, respectively. Upper and lower cases correspond to deoxyribonucleoside and ribonucleoside, respectively. 2'OMeU corresponds to 2'-O-methyluridine.

**Seq. ID**	**Sequence**	**Function**
Sequences related to PEN-DNA reactions		
α	CATTCAGGATCG	input/output exponential
aTα	A*A*G*ATCCTGAATGCGATCCTGAAT *p*	autocatalytic template
pTα	T*T*T*TTCGATCCTGAATG *p*	pseudotemplate
rTα-OG488	OregonGreen488 *A*T*TCAGAATGCGATCCTGAAT BMNQ535	reporting template
rTα-TET	TET *A*T*TCAGAATGCGATCCTGAAT BHQ1	reporting template
αbis	CATTCATCCCAG	input/output exponential
aTαbis	C*T*G*GGATGAATGCTGGGATGAA *p*	autocatalytic template
pTαbis	T*T*T*TTCTGGGATGAATG *p*	pseudotemplate
rTαbis	Atto488 *C*T*TCATGAATGCTGGGATGAAG BHQ1	reporting template
αtoω	C*A*T*TCTGGACTGAAAACAATGACTCGATCCTGA p	linear converter template
αbistoω	C*A*T*TCTGGACTGAAAAGAGAGACTCACCTGGGATGA p	linear converter template
rTω-Cy5	Cyanine5 T*T*T*TCAGTAGATGACATTCTGGACTGAAAA BHQ2	reporting template
ω	TTTTCAGTCCAGAATG	output linear
β	C*A*T*TCATCCCAG	input linear
αkβ	C*A*T*TCATCCCAGCTTCAATGACTCGATCCTGA p	killer template
pTβ	AAGCTGGGATGAATG	output killer template
βtoω	C*A*T*TCTGGACTGAAAACACGAGCTGGGATGAA p	linear converter template
let7atoα	TGCGATCCTGAAGTTTGACTCAACTATACAACCTACTACCTCA *p*	miRNA converter template
203atoα	TGCAGTCCAGAAGTTTGACTCAACTAGTGGTCCTAAACATTTCAC *p*	miRNA converter template
let7a	ugagguaguagguuguauaguu	miRNA target
miR-203a	gugaauguuuaggaccacuag	miRNA target
Sequences related to RCA reactions		
RCα	*p*CTACTACCTCACCTCAGCAAATAATTACCACAGGCAACTTCAATCCTCAGCAAATAATCAACTATACAAC	exponential RCA
RCω	*p*AACACACAACAACCCACACCGACACACAGCGCGTTTACACAACACCCACAACCAAGCAAATAATCACAG	linear RCA
α'	TGAGGATTGAAGTTGCCTGTGGTAATTATTTGC	output exponential RCA
MBω	Cyanine5 AAACGCGCAACCCACACCGACACACAGCGCG 2'OMeU 2'OMeU 2'OMeU BHQ2	reporter linear RCA
